# Downy mildew disease–suppressive soils transmit a protective core microbiome to the phyllosphere

**DOI:** 10.1093/ismejo/wrag016

**Published:** 2026-02-02

**Authors:** Jelle Spooren, Yadong Shao, Tilda Tarrant, Hannah Ploemacher, Run Qi, Syb Hopkoper, Umut G Yüce, Hangyu Dong, Pim Goossens, Saskia C M van Wees, Corné M J Pieterse, Roeland L Berendsen

**Affiliations:** Plant- Microbe Interactions, Department of Biology, Science4Life, Utrecht University, P.O. Box 800.56, 3508 TB, Utrecht, the Netherlands; Plant- Microbe Interactions, Department of Biology, Science4Life, Utrecht University, P.O. Box 800.56, 3508 TB, Utrecht, the Netherlands; Plant- Microbe Interactions, Department of Biology, Science4Life, Utrecht University, P.O. Box 800.56, 3508 TB, Utrecht, the Netherlands; Plant- Microbe Interactions, Department of Biology, Science4Life, Utrecht University, P.O. Box 800.56, 3508 TB, Utrecht, the Netherlands; Plant- Microbe Interactions, Department of Biology, Science4Life, Utrecht University, P.O. Box 800.56, 3508 TB, Utrecht, the Netherlands; Plant- Microbe Interactions, Department of Biology, Science4Life, Utrecht University, P.O. Box 800.56, 3508 TB, Utrecht, the Netherlands; Plant- Microbe Interactions, Department of Biology, Science4Life, Utrecht University, P.O. Box 800.56, 3508 TB, Utrecht, the Netherlands; Plant- Microbe Interactions, Department of Biology, Science4Life, Utrecht University, P.O. Box 800.56, 3508 TB, Utrecht, the Netherlands; Plant- Microbe Interactions, Department of Biology, Science4Life, Utrecht University, P.O. Box 800.56, 3508 TB, Utrecht, the Netherlands; Plant- Microbe Interactions, Department of Biology, Science4Life, Utrecht University, P.O. Box 800.56, 3508 TB, Utrecht, the Netherlands; Plant- Microbe Interactions, Department of Biology, Science4Life, Utrecht University, P.O. Box 800.56, 3508 TB, Utrecht, the Netherlands; Plant- Microbe Interactions, Department of Biology, Science4Life, Utrecht University, P.O. Box 800.56, 3508 TB, Utrecht, the Netherlands

**Keywords:** plant microbiomes, disease-suppressive soil, phyllosphere microbiota, microbiome assembly, downy mildew, protection

## Abstract

Plants can respond to pathogen attack by assembling disease-suppressive microbiomes. In *Arabidopsis thaliana*, infection by the obligate foliar downy mildew pathogen *Hyaloperonospora arabidopsidis* (Hpa) consistently led to the formation of a soil microbial community, referred to as “soilborne legacy” (SBL), that enhanced resistance in subsequent plant populations grown in the same soil. Previous work identified an enrichment of specific “Hpa-associated microbiota” (HAM) in the phyllospheres of infected plants, which suppressed pathogen proliferation. Here, we demonstrate how the assembly of protective HAM in the phyllosphere contributes to a disease-suppressive SBL. We identified a community of 25 core-HAM that consistently dominated the phyllospheres of 14 sets of distinct Hpa-infected plant populations across six independent experiments. Using HAM-free, gnotobiotic Hpa spores, the infection-driven assembly of a core-HAM representative was recapitulated, showing *de novo* and progressive enrichment under sustained disease pressure. Despite being transmitted via soil as SBL, HAM are phyllosphere specialists with infected leaves as their primary niche. Disease-induced HAM assembly is initiated in the phyllosphere rather than the rhizosphere, and once transmitted, they particularly accumulate on aboveground tissues. Leaf wash-offs from plant populations that inherited SBL were shown to effectively suppress downy mildew disease when applied to leaves of plants grown in unconditioned soil. These findings reveal that downy mildew disease–suppressive soils transmit a protective core microbiome to the phyllosphere, highlighting a crucial link between belowground and aboveground plant-driven microbiome assembly processes. Paradoxically, the phyllosphere thus emerges as a key assembly hub for disease-suppressive soil microbiomes.

## Introduction

Plants host diverse and complex microbiomes composed of pathogenic, commensal, and beneficial microbes [1]. Different plant tissues provide unique niches for microbial colonization [2, 3]. In the rhizosphere, the zone of soil surrounding roots, plants exude metabolites that selectively stimulate or inhibit distinct microbes [4], creating a nutrient-rich yet selective environment [5]. Some rhizosphere microbes may enter the root endosphere but must overcome additional selective pressures from root metabolites, structural root barriers, and plant immune responses [3, 6–8]. Aboveground, the phyllosphere presents a relatively nutrient-poor habitat where microbes cluster in hotspot sites that provide moisture, nutrients, or shelter from environmental stressor [3, 9–14]. Despite the distinct nature of these microbiome compartments, there is a reciprocal exchange of microbes occurring between aboveground, belowground, and inner plant tissues [10, 15, 16]. The soil, acting as a diverse microbial reservoir, exerts a major influence on the composition of plant-associated microbiomes in all compartments [16–19].

Plants can selectively steer microbial colonization to enhance their health, not only by activating immune responses to stop pathogen infection but also by stimulating protective microbiota [14, 20–22]. In response to pathogen attack, plants can selectively enrich their rhizosphere microbiome with disease-suppressive microbiota, which help reduce disease progression [14, 20, 23–26]. This phenomenon is evident in so-called disease-suppressive soils, where plants develop little disease despite the presence of virulent pathogens. Disease-suppressive soils typically emerge after an initial severe outbreak of disease, demonstrating the plant’s capacity to promote protective microbial communities in the soil [21, 27–29]. Prime examples include take-all decline in wheat and *Rhizoctonia*-suppressive soils in sugar beet, where disease suppression is linked to the buildup of disease-suppressive microbiomes in the rhizosphere and root endosphere, respectively [29–32].

The phyllosphere harbors microbiota with disease-suppressive properties that can boost plant immunity [33, 34] or act through direct microbial antagonism [33, 35]. As in soil, phyllosphere disease-suppressive microbiomes may emerge in response to pathogen infection [36, 37]. We previously discovered that phyllospheres of *Arabidopsis thaliana* (Arabidopsis) plants infected by the foliar obligate biotrophic downy mildew pathogen *Hyaloperonospora arabidopsidis* (Hpa) are enriched with a specific group of “Hpa-associated microbiota” (HAM) [38]. These HAM increase in abundance in the Hpa-infected phyllosphere environment and suppress Hpa spore production, effectively functioning as a phyllosphere disease–suppressive microbiome [38].

Previous work further demonstrated that Hpa-infected wild-type Arabidopsis Col-0 plants, referred to as a conditioning population, can condition the soil in which they grow, creating a disease-suppressive “soilborne legacy” (SBL). As a result, subsequent response populations of Arabidopsis plants grown in SBL soil reproducibly exhibit greater resistance to Hpa compared to plants grown in soil conditioned by healthy plants (control soil) [20, 38–40]. The conditioning-population plants used to generate the SBL were inoculated with either standard Hpa spore suspensions containing the HAM or with gnotobiotic, HAM-free spore suspensions (gnoHpa) [38]. In contrast, inoculation of transgenic Col-0 *RPP5* plants, resistant to Hpa infection, or mutant Arabidopsis plants deficient in *MYB72* and *F6’H1*, both essential for coumarin biosynthesis and rhizobacteria-mediated induced systemic resistance [41], did not result in a disease-suppressive SBL [38–40]. This indicates that both a successful downy mildew infection and the subsequent disease-induced plant response are necessary for the creation of disease-suppressive SBL, while the mere co-inoculation of HAM bacteria without disease induction is insufficient. Disease-suppressive HAM are enriched in the rhizosphere and phyllosphere of response population plants growing in SBL soil, even if SBL was created by conditioning population plants inoculated with HAM-free gnoHpa spores and if response population plants were not themselves infected by (gno)Hpa [38].

These insights suggest that either or both above- and belowground microbial communities could play a role in the creation and functioning of disease-suppressive soils that are triggered by and effective against foliar pathogen attack. However, the interplay between the phyllosphere and rhizosphere regarding plant-driven and disease-induced microbiome assembly processes is not well understood. Here, we tested the hypotheses that foliar downy mildew infection drives the assembly of HAM in the phyllosphere and that the soilborne transmission of these protective HAM by the disease-suppressive SBL enhances resistance of successive plant populations against Hpa. We show that disease-induced HAM assembly is initiated in the phyllosphere but that its transmission to successive plant populations proceeds via soil. These protective HAM are phyllosphere specialists that, once transmitted via the disease-suppressive SBL soil, accumulate on aboveground tissues. Plants grown in disease-suppressive SBL soil assemble phyllosphere communities that reduce downy mildew disease on naïve plants, demonstrating that HAM accumulation in the phyllosphere is key to Hpa suppression. Thus, while disease-suppressive soils are evidently often attributed to rhizosphere microbiota [27, 28], the phyllosphere emerges as a critical assembly hub for soil-transmitted disease-suppressive microbiota.

## Materials and methods

### Soil preparation, plant growth conditions, and inoculation

Field soil was collected at the Reijerscamp nature reserve in the Netherlands, where an endemic population of Arabidopsis has been found (52.0107° N, 5.7825° E) and prepared as previously described [20, 38]. Plants were sown, cultivated, and inoculated as previously described [42], with minor adaptations. In short, stratified Arabidopsis accession Col-0 seeds were sown on 120 g soil. For soilborne legacy experiments that include two growth cycles (conditioning population and response population) in the same soil, the soil surface was covered with circular cutouts of plastic micropipette-tip holders to prevent algal growth. Plants were incubated in a growth chamber (21°C, 70% relative humidity, 10-h light and 14-h dark, light intensity 100 μmol m^−2^ s^−1^) and watered twice a week with water or one-half strength Hoagland nutrient solution [43]. Two-week-old plants were either inoculated with water (mock treatment or a spore suspension of (gno)Hpa isolate Noco2 [44], as previously described [42]. Spores were collected from (gno)Hpa cultures (50–100 spores/μl) that were routinely maintained on Col-0 plants but harvested from a last passage over hypersusceptible *eds1* [45, 46] plants to proliferate sufficient pathogenic spores. (Gno)Hpa culture maintenance was performed as described [38]. Throughout this study, infected Arabidopsis phyllosphere material was collected for disease quantification 7 days postinoculation [42].

### Sample compartmentalization and genomic DNA extractions

Phyllosphere material was collected in 2 ml Eppendorf tubes by cutting the shoots with surface-sterilized razors, carefully avoiding the sampling of root or soil. No distinction between epiphytes and endophytes was made to ensure consistency with previous studies [38]. Rhizosphere soil and the root endosphere were separated as previously described [47] with minor adaptations. In brief, roots and closely adhering soil were picked from upturned pots with surface-sterilized tweezers. Roots with adhering soil were washed in 1 ml phosphate-buffered saline (PBS) buffer by gently vortexing for 5 s. Next, tubes were centrifuged for 1 min at 2350 g to spin down the rhizosphere soil while keeping the roots floating. Root material was transferred to a new tube and was washed in PBS for another five cycles. The washed-off rhizosphere soil from one sample was pooled in 15 ml Greiner tubes, vigorously vortexed, centrifuged at 4700 g for 5 min, after which the supernatant was removed. Clean roots were sonicated in PBS buffer for 5 min with 5-s pauses every 30 s, dried quickly on sterile Miracloth. Unplanted bulk soil samples were taken from the center of the pot after removing the topsoil layer (~2 cm). All samples were snap-frozen in liquid nitrogen and stored at −80°C. Two 3 mm glass beads were added to frozen phyllosphere and root endosphere samples that were mechanically lysed using the Tissuelyser II (Qiagen) for four cycles of 60 s at 30 Hz, snap freezing in between cycles. All DNA was extracted using the Qiagen MagAttract PowerSoil DNA KF Kit and a ThermoFisher KingFisher (Waltham, USA). Unplanted bulk soil, rhizosphere soil, and lysed root endosphere and phyllosphere material were suspended in 750 μl PowerMag Bead solution and spiked with *S. ruber* DNA at a concentration of 0.33% (phyllosphere, passaging experiment), 1% (bulk and rhizosphere soil, phyllosphere, microbiome compartment experiment), or 0.1% (root endosphere, microbiome compartment experiment) of the expected microbial DNA yield, determined by quantitative real-time PCR (qPCR). Suspended samples were added to the PowerMag Bead 96-well plate, and DNA was extracted according to the manufacturer’s instructions. All DNA concentrations were quantified using a NanoDrop2000.

### Hpa quantification by qPCR

For the passaging experiment, Hpa levels were quantified from genomic DNA (gDNA) extracted from the phyllosphere of (gno)Hpa, uninfected or untreated plants by qPCR in optical 96-well plates using a BioRad OPUS384 qPCR system, iTaq SYBR Green PCR Supermix (BioRad), *Arabidopsis* and Hpa actin primers, as previously described [38, 48]. In brief, reactions were run with 0.8 μM primers under a standard SYBR Green thermal profile (95°C for 10 min; 40 cycles of 95°C for 15 s, and 60°C for 1 min), followed by a melt-curve analysis. Relative Hpa abundance was calculated from ΔCt values between Hpa ACTIN and Arabidopsis ACTIN within each sample.

### 16S rRNA gene amplicon library preparation and sequencing analysis

16S rRNA gene (V3–V4 regions) amplicon sequencing libraries were prepared by Genome Quebec (Quebec, Montreal, Canada) using the NextSeq System (Illumina, 2× 300 bp paired-end sequencing, passaging experiment) or the NovaSeq System (Illumina, 2 × 250 bp paired-end sequencing, microbiome compartment experiment) [38]. Plastid- and mitochondrial-blocking peptide nucleic acids were used to prevent amplification of plant-derived sequences. 16S rRNA gene amplicon sequencing datasets included to identify core-HAM were generated as previously described [38].

Preprocessing of sequencing data was performed in the Qiime2 environment (version 2022.11) [49] and executed similarly as previously described [38]. In short, removal of primer sequences was performed using Cutadapt [50], and quality filtering, error correction, chimaera removal, and dereplication to ASVs were performed using DADA2 [51]. Optimal DADA2 truncation and maximum expected error parameters were determined using FIGARO [52]. The first five characters of the ASV identifiers were used in the text above to designate the individual ASVs. Taxonomic assignment of ASVs was performed using the VSEARCH plugin and the SILVA database (QIIME-compatible 138-release), and ASVs with unassigned taxonomies or that were assigned as Archaeal or plant derived (Chloroplast/Mitochondria) were removed. ASVs that were relatively less abundant than 0.005% (passaging experiment) or 0.00075% (microbiome compartment experiment), or that were detected in fewer than N^*^0.5 samples, were removed. Resulting datasets comprised 8 801 409 reads from 439 ASVs in 250 samples (passaging experiment) and 53 383 924 reads from 9954 ASVs in 200 samples (microbiome compartment experiment), including the spiked-in *S. ruber*. For the passaging experiment, samples “M2w5,” “M4w5,” “M9w5,” “M12w5,” “M11w4,” and “M11w5” were excluded from the analysis as qPCR showed that they were contaminated with (gno)Hpa, and sample “U2w1” was removed because of a labeling error. For the microbiome compartment experiment, samples “B-M-G2-1,” “P-M-G1-4,” “P-M-G1-3,”“E-H-G2-10,” “WR-H-G2-2,” “E-H-G2-2,” “E-M-G2-1,” “B-M-G1-4,” “E-M-G2-7,” “E-H-G2-7,” and “E-H-G2-6” were excluded from data analysis as they only had abnormally low read depth (<2500 reads, <1% of median read count per sample, similar to blank extractions), suggesting technical anomalies for those samples.

All alpha- and beta-diversity-related calculations, graphs, and differential abundance analyses were performed in R (version 3.6.3) using the Phyloseq package (version 1.30.0). All Principal Coordinate Analysis (PCoA) ordinations and Permutational Multivariate Analysis of Variance (PERMANOVA) tests were performed on Bray–Curtis dissimilarity matrices calculated for relative abundance data, using the vegan package (version 2.5.7) or vegan functionalities embedded in Phyloseq. For the microbiome compartment experiment, samples “P-M-G1–5,” “P-M-G2–4,” and “P-M-G2–2” were excluded from downstream analysis as significant outliers since they fell far outside 95% confidence intervals in ordination plots, thereby skewing multivariate analysis. PERMANOVA tests involving multiple comparisons were performed using the pairwiseAdonis package (version 0.0.4). Differential abundance testing was performed with DESeq2 [53] (package version 1.26.0) and Analysis of composition of microbiomes with bias correction (ANCOM-BC) [54] (microbiome package version 1.8.0 and nloptr package version 1.2.2.2). As we used DESeq2 to detect differences in abundance between prevalent ASVs, we used an additional prevalence filter set at 0.5^*^N. For ANCOM-BC, no additional filter step was used to enable the detection of structural zeros. ASVs associated with gnoHpa lineages in the passaging experiment were selected based on three criteria: (i) ASVs that are enriched in gnoHpa lineages compared to uninfected lineages in at least two passages from population 2 to population 5 (DESeq2 or ANCOM-BC). (ii) ASVs that are enriched in consecutive passages (populations 2, 3, 4, 5; 3, 4, 5; or 4, 5) of gnoHpa lineages compared to population 1 but not in uninfected lineages (DESeq2 or ANCOM-BC). (3) ASVs of which the relative abundance correlates to the amount of downy mildew, as quantified by qPCR, in gnoHpa and uninfected lineages (Spearman). Absolute abundances were calculated by transforming the reads of each ASV relative to the number of reads from *S. ruber*, multiplied by the amount of spiked-in DNA (ng) and the number of *S. ruber* cells expected per nanogram of *S. ruber* DNA (2.46 ^*^ 10^5^ cells/ng). Absolute abundances were corrected for plant fresh weight for the samples from the passaging experiment. For the microbiome compartment experiment, due to the sample processing, the sample fresh weight could not be obtained and uncorrected absolute abundances were used. Graphs were made using the ggplot2 (version 3.3.5), ggpubr (version 0.4.0), UpSetR (version 1.4.0), and cowplot (version 1.1.1) packages. Statistical analyses were performed using the stats (version 3.6.3) and multcompView package (version 0.1.8). Data wrangling was done with packages from the Tidyverse suite.

### Gamma-irradiated soil experiment

Gamma-irradiation (GI) of live Reijerscamp soil was performed by Steris Applied Sterilization Technologies. Sterility of GI soil was confirmed by suspending 10 g of soil in 90 ml 10 mM MgSO_4_ and plating on one-tenth-strength tryptic soy agar (1/10th TSA) medium amended with 100 mg/l cycloheximide and potato dextrose agar (PDA) medium amended with 13 mg/l chloramphenicol and 150 mg/l rose bengal before usage. Live soil, GI soil, and a 9:1 mix of live and GI soil were mixed, watered, potted, sown, and incubated in a growth chamber as described above, carefully avoiding any contact between the GI soil and the live soil. From this point onward, the GI soil was not kept in sterile conditions but was considered to have a diminished microbiome as it had a sterile starting point. The conditioning population of Arabidopsis Col-0 plants was mock- or gnoHpa-inoculated (50 spores/μl), and the response population of Arabidopsis Col-0 plants was gnoHpa-inoculated (67 spores/μl). GnoHpa was used to avoid the co-inoculation of Hpa-associated microbiota. Disease was quantified as previously described.

### Passaging experiment

Arabidopsis Col-0 plants were grown in live Reijerscamp field soil as described above but placed in Eco2Boxes (16°C, 10-h light and 14-h dark, light intensity 100 μmol m^−2^ s^−1^). Two-week-old plants were either inoculated with sterile tap water (uninfected), Hpa, or gnoHpa (80 spores/μl) or remained untreated. One week postinoculation, half of the phyllosphere, equally distributed throughout the pot, was sampled for sequencing. The other half of the plants was cut off with a razor and suspended in 1.6 ml 10 mM MgSO_4_. Leaf wash-offs were prepared by vigorously vortexing for 15 s. The leaf wash-offs were transferred to clean 2 ml perfume-spraying bottles and spray-inoculated onto a new set of 2-week-old plants. Pots were airdried and incubated in a clean set of Eco2Boxes that were labeled accordingly, so that experimental passaging lines are maintained separately. This process was repeated for a total of five successive populations. In population 5, a (gno)Hpa contamination was spotted on uninfected plants, after which the experiment was terminated. For every population, an untreated set of plants was sampled for reference. gDNA was extracted as previously described and used for (gno)Hpa disease-quantification through qPCR and sequencing. A schematic overview of this experimental setup is presented in Fig. S1.

### Microbiome compartment experiment

Live Reijerscamp field soil was conditioned by mock- and Hpa-inoculated (50 spores/μl) plants, which were grown and inoculated as described above. At the end of the conditioning population, all above-ground plant biomass was removed with a razor. Directly after, a response plant population was sown on the same soil and grown as described above. Two weeks postsowing, all plants were mock-inoculated. From the conditioning and response population, the unplanted bulk soil, roots, and phyllosphere were sampled and processed to separate the rhizosphere and root endosphere, as previously described. DNA was extracted, and samples were sent for 16S rRNA gene amplicon sequencing. A schematic overview of this experimental setup is presented in Fig. S2.

### Bacterial densities on healthy and downy mildew–infected plants

For determining phyllosphere bacterial densities on healthy and downy mildew–infected plants, plants were grown in live Reijerscamp field soil as described above and mock-, Hpa-, or gnoHpa-inoculated (50 spores/μl). Seven days postinoculation, phyllosphere material was collected in 3 ml 10 mM MgSO_4_ amended with 0.02% Silwet L77. Tubes were incubated shaking at 180 rpm for 1 h. A dilution series was prepared using 10 mM MgSO_4_, and of the 10^3^–10^7^ dilutions, 100 μl was plated on 1/10th TSA medium amended with cycloheximide and incubated at room temperature (RT). CFU numbers were quantified following 2, 4, and 6 days and normalized to shoot fresh weight. After 4 days, CFU numbers no longer increased.

### *Xanthomonas* sp. WCS2014-23 inoculation experiments

Rifampicin-resistant *Xanthomonas* sp. WCS2014-23 was cultured on selective Luria–Bertani (LB) agar (50 ng/μl rifampicin) at 28°C for 2–4 days. Single bacterial colonies were transferred to selective LB broth medium (50 ng/μl rifampicin) and cultured shaking (180 RPM) at 28°C for 1–3 days. One hundred milliliters of bacterial culture was pelleted, and bacterial cells were washed with 50 ml 10 mM MgSO_4_ three times. Optical density of the bacterial suspension was determined at 600 nm, and the *Xanthomonas* isolate was inoculated in live Reijerscamp field soil at a concentration of 10^6^ CFU/g. The soil was vigorously mixed before being potted, sown, and incubated in a growth chamber as described above. Two weeks after sowing, plants were mock inoculated with sterile tap water or inoculated with gnoHpa (50 spores/μl). For the experiment in which the *Xanthomonas* isolate was co-inoculated on the leaves with gnoHpa, plants were spray-inoculated with 1 ml of bacterial suspension (OD_600_ = 0.3), airdried, and directly after, spray-inoculated with gnoHpa (50 spores/μl). One week after inoculation, phyllosphere material was collected in 3 ml 10 mM MgSO_4_ amended with 0.02% silwet and incubated shaking (180 rpm) at RT for 1 h. A dilution series was plated on 1/10th TSA medium amended with 100 ng/μl rifampicin and 100 mg/l Delvocid, incubated at RT, and CFU were quantified 3 days after plating.

### Soilborne legacy leaf wash-off experiment

A conditioning population of Arabidopsis Col-0 plants was grown as described above and mock- or Hpa-inoculated (75 spores/μl). On the same soil, a response population of Col-0 plants was grown and mock-inoculated. Microbial leaf wash-offs were obtained by collecting all phyllosphere material in 5 ml sterilized tap water and vortexing for 15 s. One milliliter of wash-off or sterile water (Mock) was then inoculated onto 2-week-old plant populations grown as described above. Directly after, all pots were spray-inoculated with gnoHpa (30 spores/μl) and disease was quantified 7 days postinoculation.

### Statistical testing

One-sided Student’s *t*-tests were used to compare a single treatment to a control. In the case of multiple testing, *P*-values were false discovery rate (FDR)-corrected (Benjamini–Hochberg). Analysis of variance (ANOVA) with Tukey’s post hoc test was used to perform multiple comparisons of treatments to a control and among each other. Bray–Curtis dissimilarities were tested with PERMANOVA. Finally, we used DESeq2 [53] for detection of differentially abundant ASVs that are robustly present across samples and ANCOM-BC [54] for detection of ASVs that were sparsely present (structural zeros).

## Results

### Soil microbiome required for soilborne legacy

A previous finding suggested that the disease-suppressive SBL created by Arabidopsis plants infected by the foliar pathogen Hpa is the result of plant-driven infection-induced changes in the soil microbiome [20, 38, 40]. However, the evidence that disease suppression was effectuated by living microbes and not by plant-produced metabolites was not fully conclusive. To address this, Col-0 plants were grown in three soil types: (i) nonsterile field soil (100% live soil) collected at the Reijerscamp nature reserve, which harbors an abundant endemic Arabidopsis population [20], (ii) the same soil sterilized by gamma irradiation, and (iii) a 1:9 mix of live and sterilized field soil (10% live soil) [21, 32]. Plants were inoculated with gnoHpa spores or mock-treated and cultivated to condition the soil. Hereafter, a second population of Col-0 plants (response population) was sown directly in the conditioned soil and again inoculated with gnoHpa*.*

**Figure 1:**
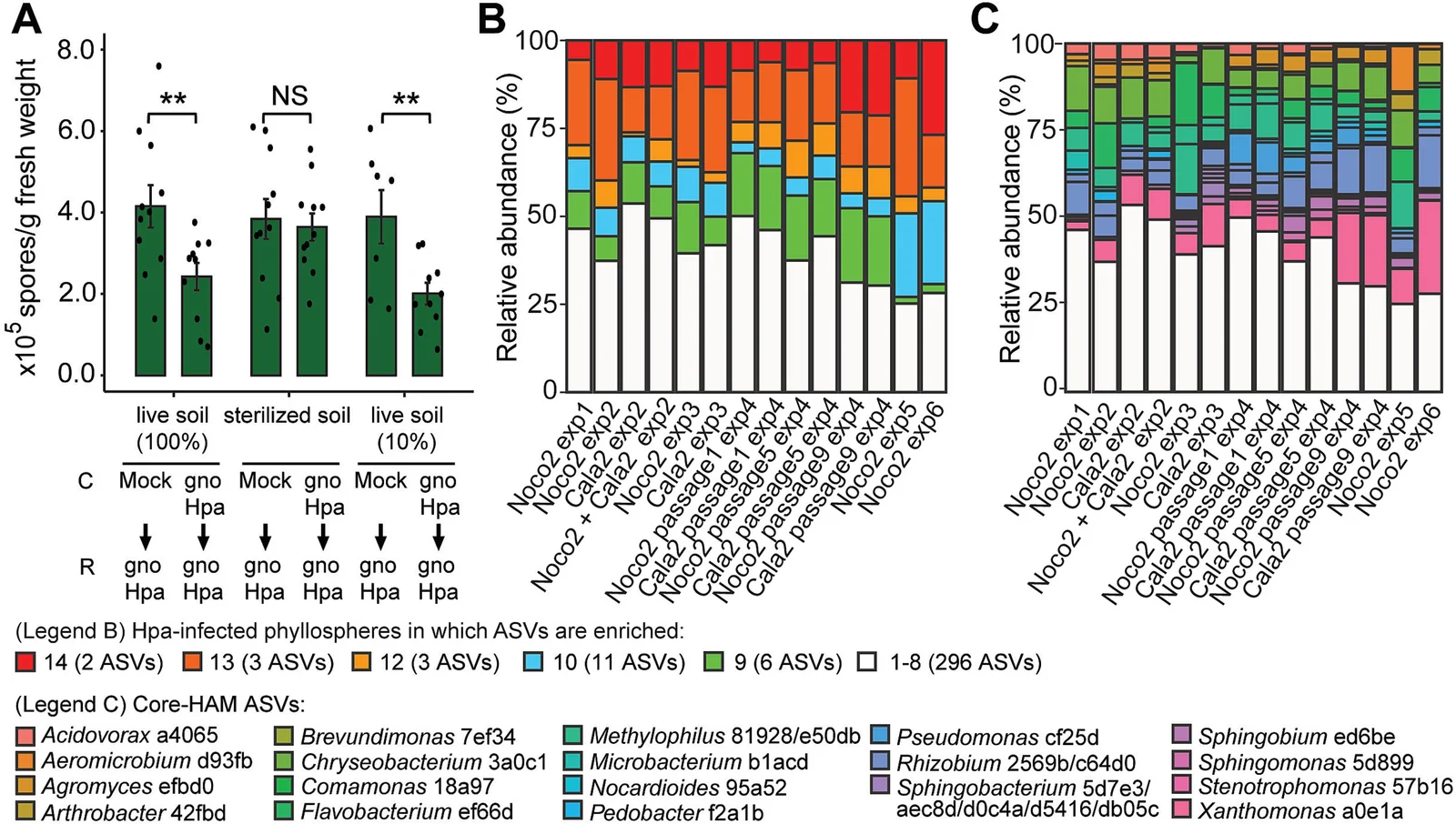
Microbial origin of the disease-suppressive SBL and relative abundance of core-HAM in Hpa-infected phyllosphere microbiomes. (A) Spore production of gnotobiotic Hpa (gnoHpa) in response (R) populations of Arabidopsis Col-0 plants growing in soil conditioned (C) by populations of Col-0 plants that were either mock-treated or inoculated with gnoHpa. Plants were grown in live Reijerscamp field soil (100% live soil), field soil sterilized by gamma-irradiation (sterilized soil), or a 1:9 mix of live and sterilized field soil (10% live soil). Asterisks indicate significance level in one-sided Student’s *t*-test, from left to right: ^**^P = .0068; NS, not significant; ^**^P = .0046. Bars and error bars indicate the average and standard error, respectively, of between 7 and 11 biological replicates. (B) Barplot showing the relative abundance (%) of the 25 core-HAM that are significantly enriched in >8 out of 14 datasets of Hpa-infected Arabidopsis phyllospheres from six independent experiments conducted over a timespan of >5 years. Core-HAM abundances were cumulated based on the number of Hpa-infected phyllospheres in which they were enriched (colors). The number of core-HAM ASVs per category is indicated in parentheses. Experiments are ordered chronologically from left to right. (C) The contribution of each single core-HAM ASV to the cumulative relative abundance (%) of the 25-member core-HAM community, colored by the taxonomy of single HAM ASVs, except for the genera *Methylophilus, Rhizobium,* and *Sphingobacterium,* which are represented by two or more ASVs.

Spore production was significantly reduced in the response population grown in live field soil conditioned by gnoHpa-inoculated plants, compared to those grown in live field soil conditioned by healthy plants (Fig. 1A). This disease-suppressive effect was lost in sterilized soil but restored by supplementation with 10% live soil, confirming a microbial origin of the SBL. These findings demonstrate that foliar Hpa infection and the resulting plant response give rise to a persistent microbial community in the soil that enhances resistance in subsequent plant populations.

### Core-HAM consistently enriched in the Hpa-infected phyllosphere

We previously showed that disease-suppressive HAM are significantly enriched in response plant populations germinating and growing in SBL soil, even when the conditioning plant population had been inoculated with HAM-free gnoHpa spores [38]. Based on this, we hypothesized that HAM, originally identified in the phyllosphere [38], are causative agents of disease suppression in the SBL. We reasoned that these microbiota could accumulate around the root systems of (gno)Hpa-infected conditioning population plants and be subsequently acquired by new plantings that germinate in the same soil. However, not all HAM that are identified as significantly enriched in Hpa-infected phyllospheres are consistently detected across experiments, suggesting some degree of context-dependent selection.

To more thoroughly define and identify HAM that are reproducibly enriched in the Hpa-infected phyllosphere compared to mock-inoculated control plants, amplicon sequencing data of 14 Hpa-infected phyllosphere datasets were analyzed. These 14 datasets were derived from six independent experiments performed over a span of 5 years. These experiments comprised Arabidopsis plants inoculated with conventionally cultured Hpa strains Noco2 or Cala2 and grown in a river sand-potting soil mixture (Exp 1 – Exp 4), or unconditioned Reijerscamp field soil (Exp 5 – Exp 6) [38]. As previous work demonstrated that Hpa infection does not significantly affect fungal communities [38]; we focused on bacterial community composition.

A total of 25 amplicon sequence variants (ASVs) representing distinct HAM were significantly enriched (*P* < .05, DESeq2, Table S1) in Hpa-infected phyllospheres compared to mock-inoculated controls in at least 9 of the 14 Hpa-infected phyllospheres. These 25 ASVs were designated as “core-HAM,” as they were consistently and reproducibly associated with Hpa (Fig. 1B and C). The 25 core-HAM ASVs accounted for 46%–75% of the bacterial abundance in the Hpa-inoculated phyllospheres but were generally low in abundance or undetectable in healthy plants (Fig. 1B, Fig. S3A). Among them, *Xanthomonas* ASV a0e1a and *Acidovorax* ASV a4065 were enriched in all tested Hpa-infected phyllospheres, occupying up to 27% relative abundance of the phyllosphere bacterial community (Fig. 1B). *Xanthomonas* ASV a0e1a was the most abundant core-HAM ASV, averaging 10% of bacterial reads from Hpa-infected leaves. Additionally, *Chryseobacterium* ASV 3a0c1, *Flavobacterium* ASV ef66d, and *Methylophilus* ASV e50db were enriched in 13 out of 14 Hpa-infected phyllospheres, whereas *Agromyces* ASV efbd0, *Pedobacter* ASV f2a1b, and *Sphingobium* ASV ed6be were enriched in 12 out of 14 Hpa*-*infected phyllospheres, respectively (Table S1, Fig. S3A). These eight highly consistent ASVs together accounted for 49% relative abundance in infected leaves while remaining low abundant (<1%) in healthy phyllospheres (Fig. S3A). These results suggest that core-HAM are natural leaf colonizers strongly promoted by the Hpa-infected phyllosphere environment. The 25 ASVs representing the core-HAM cover a broad taxonomic diversity (Fig. S3B), yet their abundance and taxonomic distribution remained stable across independent experiments conducted over a span of >5 years (Fig. 1C). This highlights the robustness of core-HAM recruitment and persistence in Hpa-infected leaves.

### Downy mildew-infected phyllospheres selectively accumulate core-HAM

Given the consistent enrichment of core-HAM in Hpa-infected phyllospheres, we hypothesized that these bacteria are selectively promoted during Hpa infection and gradually accumulate across successive infected plant populations. To test this, an initial population of Arabidopsis Col-0 plants was grown in live Reijerscamp field soil and inoculated with HAM-free gnoHpa spores. Leaf wash-offs, containing spores and associated microbiota, were then passaged to newly grown Col-0 plants in fresh live Reijerscamp soil, to generate five consecutive plant populations (gnoHpa lineages; Fig. S1). Control lineages were started by spraying the first population of plants with regular HAM-containing Hpa spore suspensions (Hpa lineages) or with a mock treatment of sterile water (uninfected lineage).

**Figure 2:**
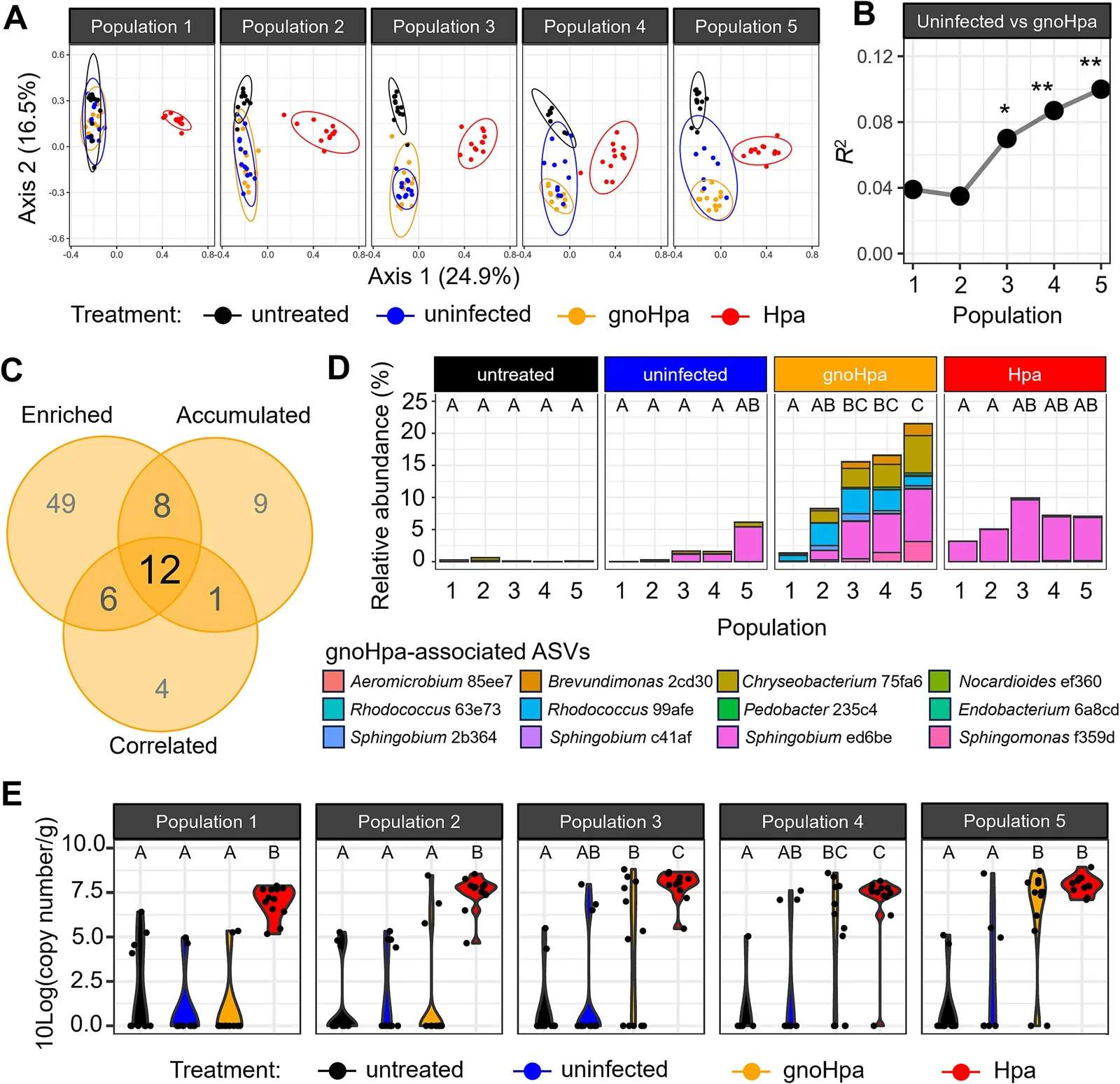
Changes in microbiome composition and enrichment of specific bacteria with persistent selective pressure in the downy mildew–infected phyllosphere. (A) Principal coordinate analysis ordination plots based on Bray–Curtis dissimilarity of phyllosphere bacterial community composition of Arabidopsis Col-0 plant populations grown in live Reijerscamp field soil. Treatments include newly grown untreated plants (black) from every population, and uninfected (blue), gnoHpa (orange), and Hpa (red) lineages in which leaf wash-offs were successively passaged from their initial inoculation (population 1) to population 5. (B) Effect size of the observed changes in microbiome composition between uninfected and gnoHpa lineages, indicated by the *R*^2^-value in PERMANOVA analysis. Asterisks indicate significance level (FDR-corrected) in PERMANOVA: (from left to right) ^*^P = .046; ^**^P = .0075; ^**^P = .0094. (C) Venn diagram showing the number of ASVs that meet each of three criteria defined to detect ASVs that were selectively promoted in gnoHpa lineages: (i) consistently enriched ASVs in gnoHpa compared to uninfected lineages in at least two out of four passages (detected by DESeq2 or ANCOM-BC), (ii) ASVs that accumulate in the gnoHpa lineages but not in the uninfected lineages (detected by DESeq2 or ANCOM-BC), and (iii) ASVs of which the relative abundances correlate with disease quantification in gnoHpa and uninfected lineages (Spearman correlations). (D) Barplots showing the cumulative relative abundance of the 12 downy mildew–associated ASVs that are identified by all three selection criteria. Relative abundances were plotted in the untreated control and uninfected, gnoHpa*,* and Hpa lineages across populations. Colors represent individual ASVs. Letters indicate significant differences (*P* < .05, ANOVA with Tukey’s *post hoc* test) in the cumulative relative abundance of all 12 ASVs tested across all populations and lineages. (E) Violin plots showing absolute abundance of core-HAM *Sphingobium* ASV ed6be, calculated based on spiked-in *S. ruber* DNA and represented as Log-10 transformed 16S copy numbers per gram of shoot fresh weight. Letters indicate significant differences (*P* < .05, ANOVA with Tukey’s *post hoc* test) tested between untreated plants and uninfected, gnoHpa*,* and Hpa lineages across populations. For all panels, each dot and bar represents one of, or the average of, 12 independent biological replicates, except for untreated population 1 (*N* = 11), uninfected population 4 (*N* = 11), and uninfected population 5 (*N* = 7).

Phyllosphere microbiome compositions were analyzed using PCoA based on Bray–Curtis dissimilarities (Fig. 2A). Consistent with previous findings [38], no significant differences in phyllosphere microbiome composition were observed between untreated, uninfected, and gnoHpa-inoculated plants in the first population (PERMANOVA results are detailed in Table S2, effect sizes are shown in Fig. S4). However, plants inoculated with HAM-containing Hpa spore suspensions exhibited a significantly distinct microbiome compared to all other lineages. In later populations, however, the phyllosphere microbiomes of the passaged uninfected, gnoHpa, and Hpa lineages all showed significant differentiation from those of untreated plants (Fig. S4, Table  S2), indicating that the transfer of phyllosphere microbial communities across successive plant populations influenced microbiome composition. By the third population, gnoHpa, and uninfected lineages also diverged significantly, with differences becoming more pronounced in the fourth and fifth populations (Fig. 2A and B, Table S2). These findings indicate that downy mildew–infected plants progressively assemble distinct phyllosphere microbiomes over time.

To identify ASVs selectively enriched in the gnoHpa lineages, we applied three filters: (i) consistent enrichment in the gnoHpa versus uninfected lineages (75 ASVs, *P*_adj_ < .05, DESeq2 or ANCOM-BC, Table S3), (ii) accumulation within the gnoHpa lineages over time but not in uninfected lineages (30 ASVs, *P*_adj_ < .05, DESeq2 or ANCOM-BC, Table S4), and (iii) positive correlation with pathogen levels, as quantified by qPCR (23 ASVs, *P*_adj_ < .05, Spearman, Table S5). We identified 12 ASVs that satisfied all three selection criteria (Fig. 2C, Tables S3–S5). Although these promoted ASVs were sporadically detected in uninfected samples, they persisted and remained abundant only in the phyllospheres of downy mildew–infected lineages after their initial appearance (Fig. S5). Their combined relative abundance increased progressively during the passages in the gnoHpa lineages and accumulated to a significantly higher cumulative relative abundance in the gnoHpa lineages compared to all other lineages (Fig. 2D).

To assess whether changes in relative abundance reflected shifts in absolute bacterial load, DNA from *Salinibacter ruber,* typically absent from soil and plant samples [55], was spiked into samples for normalization. Collectively, the 12 ASVs enriched in gnoHpa lineages were already present in relatively high numbers in population 1 of the Hpa lineages (Fig. S6), suggesting that here they constitute HAM members introduced via Hpa inoculation. In uninfected phyllospheres, these ASVs were only sporadically detected. In populations 3 and 5, cumulative abundance of the 12 gnoHpa-enriched ASVs was significantly higher compared to untreated controls that were not passaged (Fig. S6), suggesting that these are phyllosphere competent bacteria that can benefit from leaf-to-leaf transfer. Yet, their abundance was consistently significantly lower in uninfected lineages compared to Hpa-infected lineages. In the gnoHpa lineages, the absolute abundance of these 12 ASVs rose steadily with successive passages, reaching significantly higher levels compared to uninfected passaged controls and comparable to those observed in the Hpa lineages (Fig. S6). By population 5, the 12 gnoHpa-enriched ASVs were detected in high abundance in all gnoHpa-infected independent replicate lineages. These 12 downy mildew–associated ASVs thus appear to be sporadically occurring competent phyllosphere colonizers that specifically benefit from the downy mildew–infected environment and progressively build up in the phyllospheres of infected plant populations.

All 12 ASVs that accumulated in the gnoHpa lineages match genera that were previously demonstrated to be downy mildew associated [38, 56]. At the ASV level, *Sphingomonas* ASV f359d and *Brevundimonas* ASV 2 cd30 have previously been found enriched in *Peronospora effusa*-infected spinach leaves [56]. The most abundant of the 12 downy mildew–associated ASVs, *Sphingobium* ASV ed6be, is part of the core-HAM (Fig. 1B and C) and had also been found associated with *P. effusa* [56]. This ASV increased from 0.01% to 8% relative abundance between populations 1 and 5 of the gnoHpa lineages (Fig. 2D).

We further quantified the absolute abundance of *Sphingobium* ASV ed6be as 16S rRNA gene copies per gram of leaf tissue (Fig. 2E). In the Hpa lineages, ASV ed6be was abundant from the start (2.7 × 10^7^ copies/g) and increased steadily with successive passages, reaching 1.5 × 10^8^ copies/g in population 5. In the gnoHpa lineages, the prevalence of ASV ed6be rose across passages, being detected in 2, 3, 7, 8, and 10 out of 12 lineages from population 1 to 5, respectively. Its average abundance increased 2556-fold, from 3.3 × 10^4^ to 8.5 × 10^7^ copies/g. In contrast, ASV ed6be was only sporadically detected in uninfected lineages (1–4 out of 12 lineages per population) and remained low in abundance. Thus, although this ASV can occur in uninfected phyllospheres, it persists and proliferates specifically under the selective regime imposed by downy mildew infection.

Collectively, our data demonstrate that among the diverse microbiota capable of colonizing plants grown in live Reijerscamp field soil, distinct downy mildew–associated bacteria including core-HAM become consistently assembled in the phyllosphere of downy mildew–infected plants.

### Core-HAM are phyllosphere specialists that are inherited as soilborne legacy

A live soil microbiome is required for the creation of an SBL by Hpa-infected plants (Fig. 1A), and previous findings have suggested that HAM bacteria are assembled in both the rhizosphere and phyllosphere of response population plants grown in SBL soil [38]. This supports the idea that core-HAM originates from the microbial community in the SBL soil conditioned by (gno)Hpa-infected plants and subsequently forms a disease-suppressive microbiome on a next generation of plant hosts. The root endosphere may serve as a conduit for microbial migration between belowground and aboveground habitats [15]. However, the connection between core-HAM colonization in the rhizosphere, root endosphere, and phyllosphere of Hpa-infected plants and their establishment in successive plant populations grown in SBL soil remains unclear.

To trace the origin and spatiotemporal colonization of core-HAM in Col-0 plants grown in the disease-suppressive SBL soil, we generated a detailed map of the microbiota communities in unplanted bulk soil, rhizosphere, root endosphere, and phyllosphere in both conditioning and response plant populations in an SBL experiment (Fig. S2). Bacterial community compositions in all microbiome compartments were characterized using 16S amplicon sequencing, enabling comparisons of identity and abundance across spatial compartments and time. Microbiomes in bulk soil, rhizosphere, root endosphere, and phyllosphere were significantly distinct, confirming successful separation of these microbiome compartments (PERMANOVA results are detailed in Tables S6 and S7).

We detected a total of 9953 distinct ASVs (Fig. S7). Of the ASVs detected in rhizosphere, root endosphere, and phyllosphere, respectively, 99%, 97%, and 94%, were also detected in unplanted bulk soil. These ASVs cumulatively accounted for over 98% of the relative abundance in the rhizosphere and root endosphere and 83% in the phyllosphere. Even in the aboveground phyllosphere, 98% of the community’s relative abundance comprised bacteria also detected belowground. This supports the notion that the soil acts as a microbial reservoir for the assembly of plant-associated microbial communities, including those in the aboveground phyllosphere environment [16, 17, 19].

**Figure 3:**
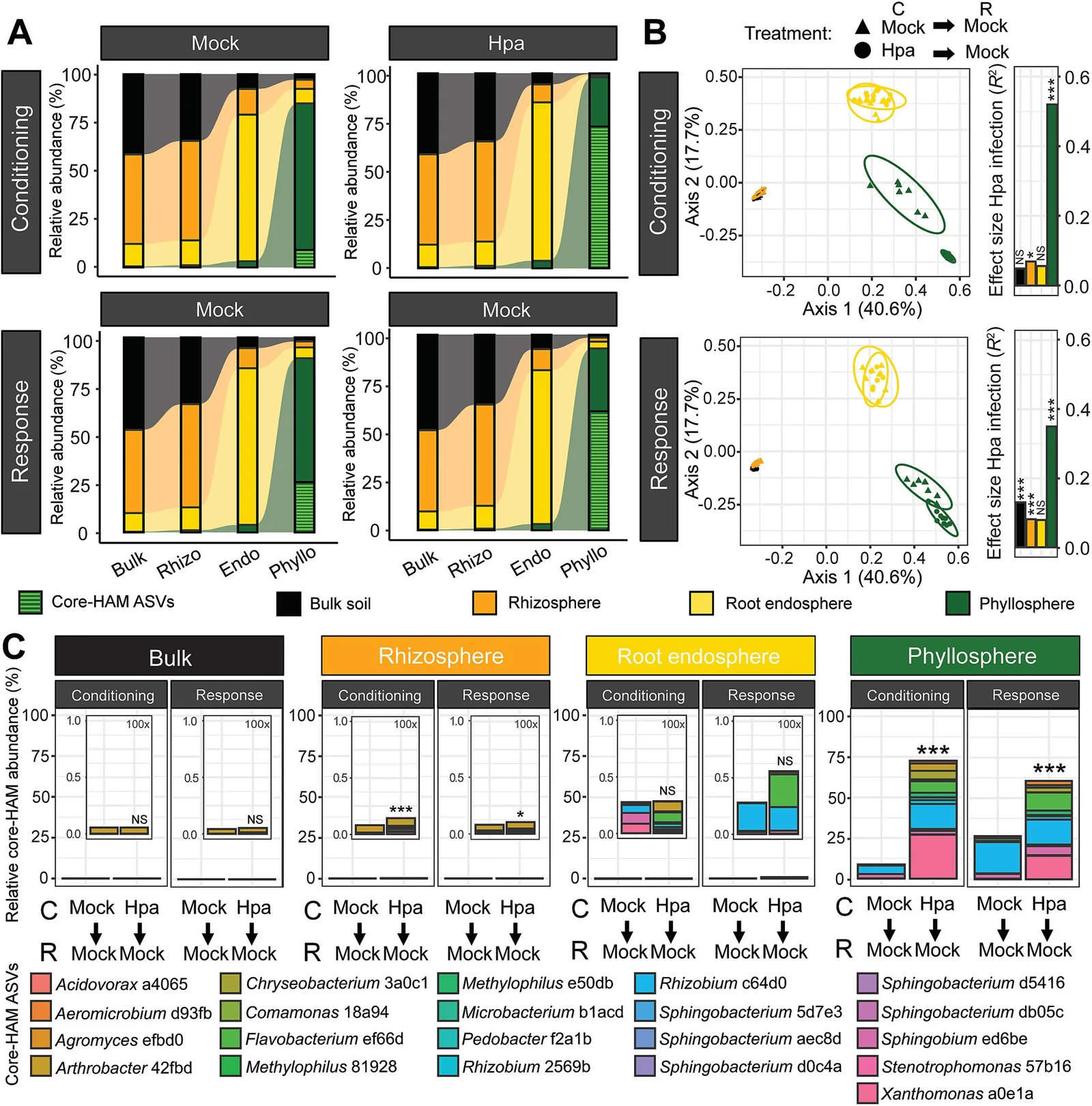
Spatiotemporal dynamics of microbiota in distinct microbiome compartments from Hpa-infected plants and healthy plants grown in disease-suppressive SBL soil. Arabidopsis Col-0 plants were grown as mock- or Hpa-inoculated conditioning plant population (C) or as mock-inoculated response plant population (R) grown in disease-suppressive SBL soil conditioned by Hpa-inoculated plants or control soil conditioned by mock-inoculated plants (Fig. S2). Microbiome composition in the unplanted bulk soil (black), rhizosphere (orange), root endosphere (gold), and phyllosphere (dark green) microbiome compartments of these plants was analyzed with 16S amplicon sequencing. All data are based on 6–10 biological replicates per microbiome compartment per plant population. (A) Sankey plot showing the contribution of ASVs with the highest relative abundance in each microbiome compartment relative to the total relative abundance within each microbiome compartment. Light-green dashed lines indicate the relative abundance of the 21 core-HAM ASVs across the microbiome compartments in the experiment. (B) Principal coordinate analysis ordination plot based on Bray–Curtis dissimilarities showing bacterial community composition in the microbiome compartments of mock- (triangles) or Hpa-inoculated (circles) plants. The conditioning population (C) plants were either mock- or Hpa*-*inoculated, and the response population (R) plants were mock inoculated but grown in soil conditioned by either mock- or Hpa-inoculated plants. Bars indicate effect size (*R*^2^) based on PERMANOVA analysis, representing the impact of Hpa infection on microbiome community composition in the conditioning population plants or the corresponding SBL effect in the response population plants. Asterisks indicate significance level (FDR-corrected) in PERMANOVA: NS, not significant; ^*^P = .011; ^***^P < .001. (C) Barplots representing the average cumulative relative abundances (%) of the 21 core-HAM ASVs in each microbiome compartment per plant population. For belowground microbiome compartments, inner panels show 100× magnification of the outer panel, with relative abundances on the *y*-axis scaled from 0.0 to 1.0%. Colors represent individual core-HAM ASVs. Asterisks indicate significance level of the Hpa infection on core-HAM relative abundance in the conditioning population plants or on the corresponding SBL effect in the response population plants in FDR-corrected one-sided Student’s *t-*test (left to right): NS, not significant; ^***^P = 8.4 10^−4^; ^*^P = .046; ^***^P = 2.1 × 10^−10^; ^***^P = 1.4 × 10^−6^.

We assigned each of the 9953 detected ASVs to the compartment in which it reached its highest relative abundance, resulting in 3936 “bulk soil” ASVs, 4119 “rhizosphere” ASVs, 1553 “root endosphere” ASVs, and 345 “phyllosphere” ASVs. As expected, the ASVs with high relative abundance in the bulk soil and rhizosphere categories are numerous and taxonomically diverse, with seven bacterial phyla representing >1% relative abundance (Fig. S8). In contrast, only a select number of ASVs in the root endosphere and phyllosphere reach the same threshold of 1% relative abundance, suggesting strong niche specialization. In this light, Fig. 3A shows that the root endosphere and especially the phyllosphere compartment favor the growth of a select group of microbiota. For example, the 345 “phyllosphere” ASVs, which had the highest relative abundance in the phyllosphere microbiome compartment, represent only 12% of the total number of ASVs detected in the phyllosphere but accounted for 85% of the total relative abundance in this compartment. In contrast, the cumulative relative abundance of all “phyllosphere” ASVs combined in the bulk soil was below 1%. These results demonstrate that microbiota originating from the bulk soil thrive best in distinct ecological niches provided by the plant, with strong selective effects observed in the root endosphere and the phyllosphere.

We investigated whether the effect of downy mildew infection on microbiome composition was also compartment specific (Fig. 3B, Table S8). Consistent with earlier findings [38, 40] (Fig. 2), Hpa infection significantly altered rhizosphere and phyllosphere communities in conditioning population plants. No significant effects were observed in bulk soil or root endosphere. Also in response populations, noninfected plants grown in disease-suppressive SBL soil exhibited distinct rhizosphere and phyllosphere microbiomes compared to those grown in control soil conditioned by mock-treated plants. The effect of SBL on microbiome composition was significant yet modest in the rhizosphere of response population plants (*P* < .001, *R*^2^ = 0.081 in PERMANOVA) but more pronounced in the phyllosphere (*P* < .0001, *R*^2^ = 0.35 in PERMANOVA). This paradoxically suggests that a belowground microbial legacy most strongly affects the aboveground microbiome of a subsequent planting.

Of the 25 core-HAM ASVs identified earlier (Fig. 1B and C), 21 ASVs were also detected in this experiment. These 21 ASVs were all detected in the phyllosphere. In belowground plant compartments, 15 core-HAM ASVs were detected in the rhizosphere, 10 in the root endosphere, but only 8 in bulk soil (Table S9). This either suggests that the majority of core-HAM have abundances below the detection limit in bulk soil and that their competitive colonization is particularly favored on or within the plant host or that they originate from alternative sources. The collective abundance of the 21 core-HAM ASVs was low in the bulk soil, rhizosphere, and root endosphere (<1.0%). Cumulative core-HAM ASV relative abundances significantly increased in both the rhizospheres of Hpa-infected conditioning population plants and of mock-treated response population plants grown in SBL soil but not in the bulk soil or root endosphere of these plants (Fig. 3C).

All 21 core-HAM ASVs reached the highest relative abundances in the phyllosphere. Consistent with previous findings, they accounted for a high relative abundance (73%) in the Hpa-inoculated phyllosphere of conditioning population plants compared to mock-inoculated control plants (9%, Fig. 3C). Even in healthy response plants grown in SBL soil, the relative abundance of the 21 core-HAM ASVs was significantly higher in the phyllosphere of plants grown in disease-suppressive SBL soil (61%) compared to plants grown in control soil conditioned by healthy plants (27%; Fig. 3C). Thus, the microbial SBL created by Hpa-infected plants drives a pronounced shift in the phyllosphere microbiome of subsequent plantings, which become dominated by core-HAM.

To determine whether this enrichment reflected increased bacterial population densities or displacement of phyllosphere resident microbiota, we quantified absolute abundances in each microbiome compartment using spiked-in *S. ruber* DNA. Hpa infection of conditioning population plants significantly increased total bacterial load in both phyllosphere and rhizosphere (Fig. S9). Also, response population plants grown in SBL soil had significantly higher bacterial loads than those in control soil but only in the phyllosphere. This increase corresponded with the elevated absolute abundance of core-HAM, whereas the absolute abundance of other ASVs remained unchanged (Figs S9 and S10). These results confirm that the microbiomes of plants grown in SBL soil are primarily affected aboveground, where especially their phyllospheres become more densely colonized by disease-suppressive HAM. This raises the question of whether the enrichment of soilborne HAM occurs primarily, and perhaps exclusively, in the phyllosphere, rather than requiring substantial migration from belowground compartments.

### Downy mildew infection enriches a soilborne core-HAM isolate specifically in the phyllosphere

Previous work showed that HAM ASVs are enriched in both the rhizosphere and phyllosphere of plants grown in disease-suppressive SBL soil and that their assembly is promoted by downy mildew infection [38]. However, although these ASVs originate from soil and contribute to the disease-suppressive legacy, they paradoxically accumulate most prominently in the phyllosphere of plants grown in SBL soil (Fig. 3). This observation led us to hypothesize that the disease-induced assembly of core-HAM, which appears to be causal for the creation of downy mildew disease–suppressive soils, is initiated in the phyllosphere rather than the rhizosphere.

**Figure 4:**
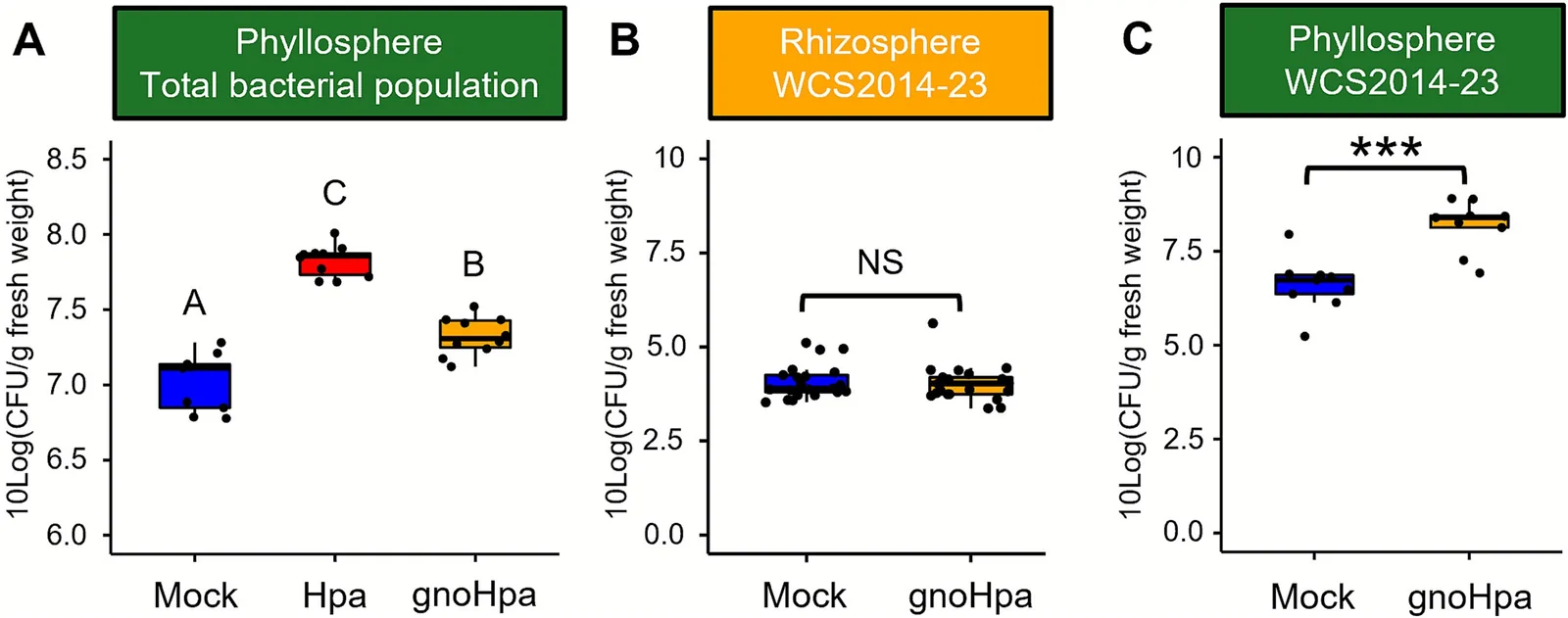
Total bacterial abundances and core-HAM *Xanthomonas* sp. WCS2014-23 population densities on downy mildew–infected plants. (A) Boxplots showing phyllosphere bacterial population densities (10-log transformed CFU/g shoot fresh weight) of mock-, Hpa-, or gnoHpa-inoculated Arabidopsis Col-0 plant populations grown in live Reijerscamp field soil. Letters indicate significance based on the average of 10 biological replicates (*P* < .05 in ANOVA with Tukey’s *post hoc* test). (B, C) Boxplots showing population densities (10-log transformed CFU/g fresh weight) of *Xanthomonas* sp. WCS2014-23, which was inoculated into live Reijerscamp field soil, in the (B) rhizosphere and (C) phyllosphere of mock- and gnoHpa-inoculated Arabidopsis Col-0 plant populations. Asterisks indicate significance level based on one-sided Student’s *t*-test of 20 (rhizosphere) and 9 (phyllosphere) biological replicates: NS, not significant; ^***^P = 1.0 × 10^−4^.

Downy mildew infection coincides with an increased bacterial load in the phyllosphere (Fig. 2E, Fig. S9). To confirm this, we quantified bacterial colony-forming units (CFU) in the phyllosphere of Arabidopsis Col-0 plants grown in live Reijerscamp field soil that were either mock-treated or inoculated with HAM-containing Hpa or HAM-free gnoHpa spore suspensions. In both Hpa- and gnoHpa-inoculated plants, phyllosphere bacterial densities were significantly higher than in mock-treated controls (Fig. 4A), indicating that downy mildew infection, and not just the co-inoculation of HAM, promotes the proliferation of phyllosphere-associated bacteria.

To investigate whether core-HAM are specifically promoted in the phyllosphere, we selected *Xanthomonas* sp. WCS2014-23, which was isolated from the roots of Hpa*-*infected Arabidopsis plants [20] and is represented by the most abundant and robust core-HAM *Xanthomonas* ASV a0e1a. Rifampicin-resistant WCS2014-23 was mixed into live Reijerscamp field soil at 10^6^ CFU/g soil, and Col-0 plants were grown in this soil. Two-weeks-old plants were mock-treated or inoculated with gnoHpa, and at 7 days postinoculation, densities of WCS2014-23 were quantified in both the rhizosphere and phyllosphere. Despite being introduced via the soil, WCS2014-23 population densities were ~1000-fold higher in the phyllosphere than in the rhizosphere (Fig. 4B and C). Rhizosphere colonization was unaffected by infection status, whereas phyllosphere colonization was significantly higher in gnoHpa-infected plants (Fig. 4B and C). When co-inoculated directly into the phyllosphere with gnoHpa, WCS2014-23 populations reached densities of 10^8.8^ CFU per gram of diseased leaf tissue, compared to 10^7.1^ CFU per gram of leaf tissue of healthy plants (Fig. S11). These results demonstrate that, even though core-HAM *Xanthomonas* sp. WCS2014-23 originates from the soil, it preferentially colonizes the phyllosphere, where its growth is strongly promoted by downy mildew infection. Future work will be needed to determine whether, in addition to the representative example of *Xanthomonas* sp. WCS2014-23, other core-HAM taxa show similar enrichment patterns.

### Accumulation of Hpa-associated microbiota in the phyllosphere suppresses downy mildew disease

**Figure 5:**
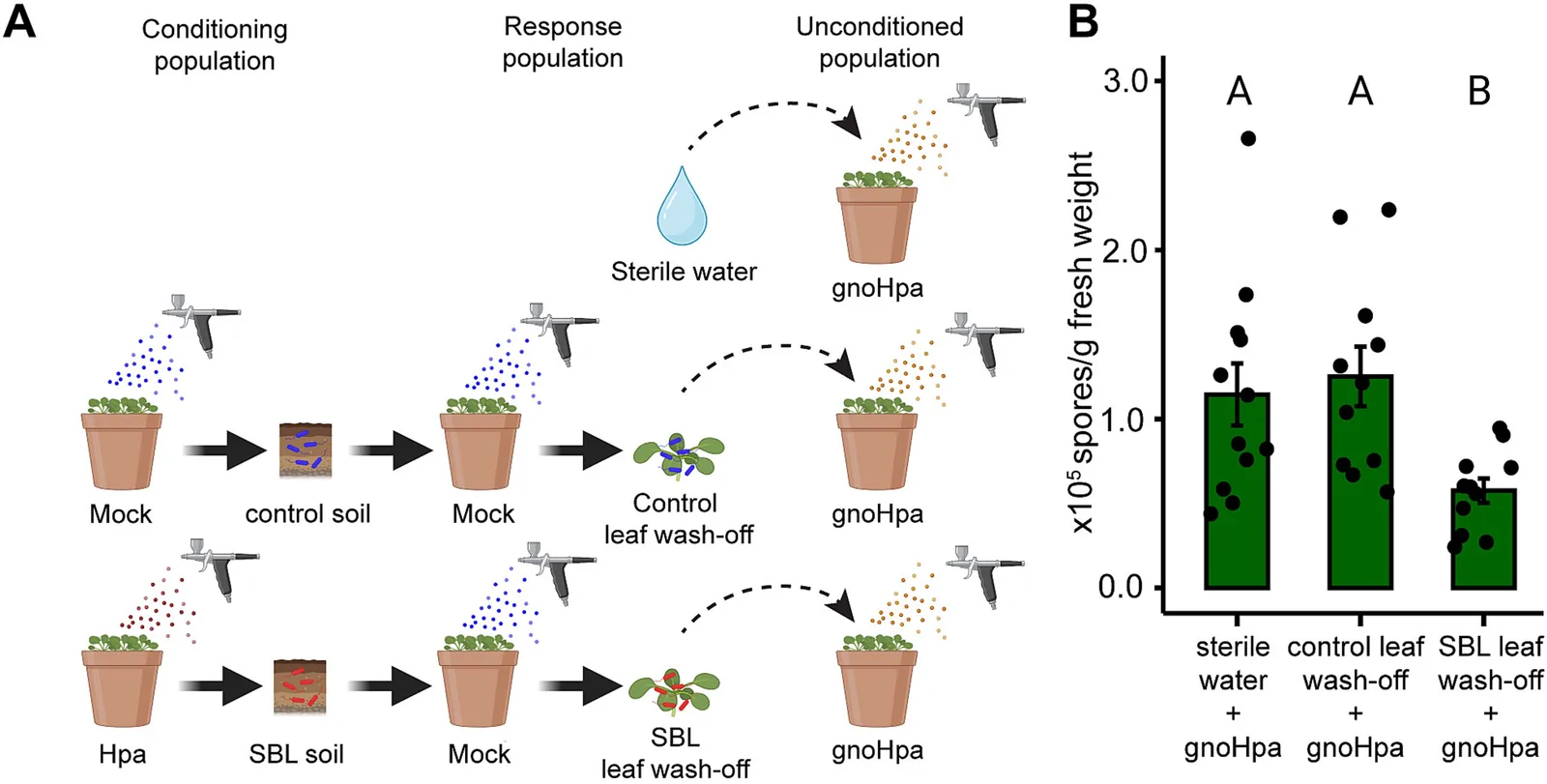
Spore production on Arabidopsis Col-0 plants inoculated with gnoHpa spores in presence of microbial leaf wash-offs from plants grown in disease-suppressive SBL soil or control soil. (A) Schematic representation of the experimental setup used to test the effect of phyllosphere microbiomes on subsequent downy mildew infection. Conditioning populations of Arabidopsis Col-0 plants were grown in live Reijerscamp field soil and either mock or Hpa inoculated. After 1 week, the conditioning population plants were removed, and new Col-0 plants (response population) were sown and grown in the soils conditioned by healthy plants (control soil) and soil conditioned by Hpa-infected plants (SBL soil). Response population plants were mock inoculated. Microbial leaf wash-offs were collected from healthy plants grown in control or the disease-suppressive SBL soils and mixed with gnoHpa spores before being used to inoculate a new set of Col-0 plants grown in unconditioned soil. GnoHpa spores suspended in sterile water were used as the control inoculum. Spore production was quantified 7 days postinoculation. (B) Barplot showing the average spore production on a population of Arabidopsis Col-0 plants grown in unconditioned live Reijerscamp field soil inoculated with a suspension of gnoHpa spores in either sterile water or microbial leaf wash-offs from a healthy response plant population grown in soil conditioned by mock- (control leaf wash-off) or Hpa-inoculated (SBL leaf wash-offs) plant populations. Error bars represent standard error based on 11–12 biological replicates; letters indicate significant differences (*P* < .05 in ANOVA with Tukey’s *post hoc* test).

Previous work demonstrated that direct co-inoculation of HAM with gnoHpa in the phyllosphere suppresses downy mildew spore formation [38]. We thus wondered whether the HAM community that naturally assembles in the phyllosphere of healthy plants grown in disease-suppressive SBL soil similarly provides protection against a subsequent downy mildew infection. To test this, we used our standard SBL setup (Fig. S2), conditioning Reijerscamp field soil with either Hpa- or mock-inoculated Col-0 plants. Healthy Col-0 response populations were then grown in the conditioned soils. This design allowed HAM-enriched phyllosphere communities to assemble on plants in SBL soil while avoiding any confounding effects of ongoing Hpa infection. Microbial leaf wash-offs were collected from both groups, mixed with HAM-free gnoHpa spores, and used to inoculate a third set of Col-0 plants grown in unconditioned live soil. Disease severity was quantified as spore production 7 days postinoculation (Fig. 5A).

Bacterial leaf wash-offs from plants grown in control soil had no impact on downy mildew disease development, as the spore production on plants inoculated with gnoHpa mixed with control leaf wash-off was similar to plants inoculated with gnoHpa spores suspended in sterile water (Fig. 5B). In contrast, when gnoHpa spores were mixed with the HAM-enriched bacterial leaf wash-offs from plants grown in disease-suppressive SBL soil, the spore production was significantly reduced. This demonstrates that the phyllosphere microbiome assembled in healthy plants grown in disease-suppressive SBL soil confers protection against downy mildew. These findings indicate that the suppressiveness of SBL soil indeed results from the soilborne inheritance of HAM. The assembly of HAM in the phyllosphere of successive plant populations provides effective protection against downy mildew disease. The mechanism by which this protection is effectuated (e.g. induction of plant immune responses, direct microbe–microbe interactions, metabolite production) requires further investigation.

## Discussion

Both rhizosphere and phyllosphere microbiomes are crucial to sustain plant health [57, 58]. It is well established that the assembly of beneficial microbiota belowground can give rise to disease-suppressive soils [14, 27, 28]. In such soils, plants are protected against pathogens either by rhizobacteria-mediated production of antibiotics [21, 30], competition over scarce resources (e.g. iron) [59, 60], or via the induction of systemic resistance [61, 62]. Although research on disease-suppressive soils has largely focused on their effects against root pathogens [27, 29, 32], disease-suppressive soils can also protect from pathogens in aboveground plant parts [14, 15, 34, 38]. Here, we demonstrated that downy mildew–infected plants assemble a protective core microbiome in the phyllosphere and that these disease-suppressive microbiota subsequently form an SBL that is transmitted to the phyllosphere of successive plant populations grown in the same soil. This indicates that the phyllosphere can act as a key assembly hub for soilborne disease-suppressive microbiomes.

The diverse soil microbiome serves as the main microbial reservoir from which the phyllosphere microbiome is assembled [16, 18, 19]. Our results confirm that the majority of phyllosphere microbiota are also present in the soil and are likely to originate from it. However, despite the significant taxonomical and functional overlap between rhizosphere and phyllosphere microbiota [17], our data suggest that the soilborne phyllosphere microbiota are specialized to thrive on aboveground plant tissues. Among these phyllosphere specialists are the core-HAM, which seem particularly well adapted to the selective environment imposed by downy mildew infections, facilitating their consistent and specific assembly on diseased plants.

The mechanisms underlying core-HAM assembly in the Hpa-infected phyllosphere remain to be elucidated. Both the nutrient environment provided by the leaf surface and the plant’s immunological status are known drivers of phyllosphere microbiome assembly [63, 64]. In line with this, we previously showed that fully resistant Col-0 RPP5 plants and coumarin-biosynthesis mutants do not generate a disease-suppressive SBL when inoculated with HAM-containing Hpa spores [38, 40], indicating that HAM assembly and transmission are at least partly governed by plant-dependent processes. We also demonstrated that core-HAM abundances, initially high after infection, decline once Hpa is removed and leaf wash-offs are serially passaged [38]. Together, these findings support the hypothesis that core-HAM are specialized to exploit the transient niches created by the infection-induced changes in leaf nutrient availability and plant immune responses.

Our finding that phyllosphere microbiomes of plants grown in SBL soil are strongly affected by the soil community is consistent with previous work showing that phyllosphere microbial communities are shaped by and differentiate from the soil microbiome [16, 19]. We hypothesize that the initial infection-induced assembly of disease-suppressive core-HAM in the phyllosphere (Figs 2 and 4) is followed by their buildup in disease-suppressive SBL soil (Fig. 3) and that they then reassemble in the phyllosphere of successive plant populations to protect against Hpa (Figs 3–5). The endosphere has been identified as a potential route for bidirectional microbial migration between the rhizosphere and the phyllosphere [15] and an important compartment for microbial legacies [65]. However, our data (Fig. 3, Table S9) suggest that the endosphere is of minor importance for the migration of core-HAM. Firstly, only a subset of core-HAM colonizes the root endosphere. Secondly, core-HAM abundances did not increase significantly in either the root endosphere of Hpa-infected plants or plants grown in the disease-suppressive SBL soil. Thirdly, certain core-HAM that accumulated in the phyllosphere of plants grown in SBL soil were not detected in the endophytic compartment. This suggests that core-HAM are likely transmitted through alternative routes. One possible alternative could be that priority effects [66–68] and subsequent host filtering [19] allow the phyllosphere competent HAM in disease-suppressive SBL soil to lift and preferentially colonize the new plant shoots as they emerge [69]. The spatiotemporal dynamic of HAM assembly and transmission could be addressed with a tissue-segmented time-series amplicon sequencing approach on Hpa-infected conditioning population plants or response population plants grown in SBL soil.

Although the infection-induced assembly of disease-suppressive microbiota has been separately documented to occur in both the rhizosphere and phyllosphere [20, 21, 23, 24, 36, 37], research on disease-suppressive soils has evidently predominantly concentrated on the rhizosphere due to its direct interface with plant roots. However, our results provide evidence of a critical link between belowground and aboveground disease-suppressive microbiome assembly processes with a crucial role of phyllosphere microbiomes in the functioning of downy mildew disease–suppressive soils. Uncovering the mechanisms by which phyllosphere microbiota are assembled, persist, and transmitted, particularly in pathogen-stressed environments, could provide novel strategies for biocontrol and sustainable crop protection. We successfully recapitulated the recruitment of the core-HAM *Sphingobium* ASV ed6be by inoculating Arabidopsis plants grown in field soil with HAM-free gnoHpa spores and serial passaging of phyllosphere leaf wash-offs. *Sphingobium* ASV ed6be was also enriched on laboratory-grown spinach leaves infected by the downy mildew *P. effusa*, whereas various other gnoHpa-enriched ASVs (Fig. 2) were enriched on naturally *P. effusa* infected spinach grown in a commercial field [56]. This indicates that phyllosphere abundance of protective core-HAM could be translated to a commercially relevant crop and that the selective enrichment of specific microbes in laboratory cultures could reflect infected field conditions. To harness the plant-beneficial potential of such microbiota, future research should focus on characterizing the phyllosphere metabolic landscape, the plant immunological pathways and the bacterial traits that drive the assembly of disease-suppressive microbiomes during pathogen attack.

Based on the infection-induced assembly of protective core-HAM, their soilborne transmission to the phyllosphere of plants grown in downy mildew disease–suppressive soils, and their ease of spreading when washed off from leaves [38], we hypothesize that successive infections in agricultural fields could create a feed-forward loop leading to the progressive suppression of disease. The phyllosphere might serve as a crucial assembly and distribution hub from which disease-suppressive microbiomes—both rhizosphere and phyllosphere-associated—can disseminate throughout plant populations, potentially leading to fieldwide disease suppressiveness. This revelation raises an important question: could the phyllosphere be involved in other types of disease-suppressive soils, including those that are of agricultural relevance? Evidence supports the possibility of taxonomical and functional overlap between microbes that suppress both soilborne and foliar pathogens. For example, beneficial pseudomonads, which are known to antagonize the soilborne pathogen *Gaeumannomyces tritici* in take-all decline soils of wheat [30, 31], have also been implicated in the suppression of the foliar pathogen *Zymoseptoria tritici* in the wheat phyllosphere [35]. Similarly, a beneficial *Streptomyces* sp., initially identified for its ability to suppress Fusarium wilt disease in the rhizosphere of strawberry plants [70], can migrate bidirectionally throughout the plant endosphere and vasculature bundles, increasing resistance against *Botrytis cinerea* infections in the strawberry phyllosphere [15]. Thus, the phyllosphere microbiome may indeed be a crucial component of disease-suppressive soils of agricultural relevance that has been largely overlooked in past research and warrants further investigation.

## Supplementary Material

Supplemental_Spooren_et_al_2026_ISMEJ_accepted_wrag016

## Data Availability

The experimental data and the postprocessing amplicon sequencing data that support the findings of this study are available at https://github.com/JelleSpooren/Spooren-et-al-2025, together with the code used to analyze the data and generate figures. Raw amplicon sequence data generated by this study are available at https://www.ncbi.nlm.nih.gov/sra/PRJNA1262419.
