# Supplementary material for: Downy mildew disease–suppressive soils transmit a protective core microbiome to the phyllosphere
Source: ISME J. 2026 Feb 2;20(1):wrag016. doi: 10.1093/ismejo/wrag016 (PMC12927876; doi:10.1093/ismejo/wrag016)
Supplement: Supplemental_Spooren_et_al_2026_ISMEJ_accepted_wrag016 [file supplemental_spooren_et_al_2026_ismej_accepted_wrag016.docx]

## **Supporting Information**

**Article title**: Downy mildew disease-suppressive soils transmit a protective core microbiome to the phyllosphere

**Authors:** Jelle Spooren, Yadong Shao, Tilda Tarrant, Hannah Ploemacher, Run Qi, Syb Hopkoper, Umut Güney Yüce, Hangyu Dong, Pim Goossens, Saskia C.M. van Wees, Corné M.J. Pieterse, Roeland L. Berendsen

**The following Supporting Information is available for this article:**

Supplementary Figures

**Figure S1. Passaging experimental setup.**

**Figure S2. Microbiome compartment experimental setup.**

**Figure S3. Abundance and taxonomic diversity of core-HAM ASVs in Hpa-infected and healthy plants across 6 independent experiments.**

**Figure S4. Changes in phyllosphere microbiome composition in passaged lineages compared to untreated control plants.**

**Figure S5. Relative abundance of the 12 gnoHpa-associated ASVs per lineages.**

**Figure S6. Absolute abundances of the 12 gnoHpa-associated ASVs.**

**Figure S7. The total of 9953 ASVs and their detection per microbiome compartment.**

**Figure S8. Taxonomic diversity of ASVs that were most abundantly detected in either bulk soil, rhizosphere, root endosphere or phyllosphere microbiome compartments.**

**Figure S9. Absolute abundances of the total bacterial communities and core-HAM in the microbiome compartment experiment.**

**Figure S10. Absolute abundances of all ASVs that are not part of the core-HAM community in the microbiome compartment experiment.**

**Figure S11. Bacterial population densities of *Xanthomonas* isolate WCS2014-23 when co-inoculated with gnoHpa directly in the phyllosphere.**

Supplementary Tables

**Table S1: Core-HAM ASVs that were enriched (FDR-corrected Wald-test, DESeq2) in more than 8 out of 14 Hpa-cultures tested across 6 independent experiments that were performed in a time span of over 5 years.**

**Table S2: PERMANOVA (permutations = 9999) results of pairwise comparisons between uninfected, Hpa and gnoHpa lineages and untreated control plants per population in the passaging experiment.**

**Table S3: ASVs that were enriched (ANCOM-BC or DESeq2, FDR*-*corrected *P* value < 0.05) in gnoHpa-infected lineages compared to uninfected lineages in at least 2 populations of populations 2-5.**

**Table S4: ASVs that were enriched (ANCOM-BC or DESeq2, FDR*-*corrected *P* value < 0.05) in successive populations within gnoHpa-lineages compared to population 1, but not in uninfected-lineages.**

**Table S5: ASVs that correlate (spearman, FDR-corrected *P* value < 0.05, indicated by ‘*P*_adj_’) with gnoHpa disease-quantification by qPCR compared to uninfected-lineages. *R*^2^ is a measure for effect size.**

**Table S6: PERMANOVA (permutations = 9999) of factors ‘Compartment’ * ‘Treatment’ * ‘Generation’ on Bray-Curtis dissimilarities.**

**Table S7: PERMANOVA (permutations = 9999) results of pairwise comparisons between distinct microbiome compartments.**

**Table S8: PERMANOVA (permutations = 9999) results of treatment effect (Mock vs Hpa) within microbiome compartments in conditioning population plants and response population plants.**

**Table S9: Core-HAM ASVs and detection (+/-) in the phyllosphere, rhizosphere, root endosphere and bulk soil Taxonomy indicates genus or family level of each ASV. ASVs are depicted by the first five characters of their identifiers.**

**
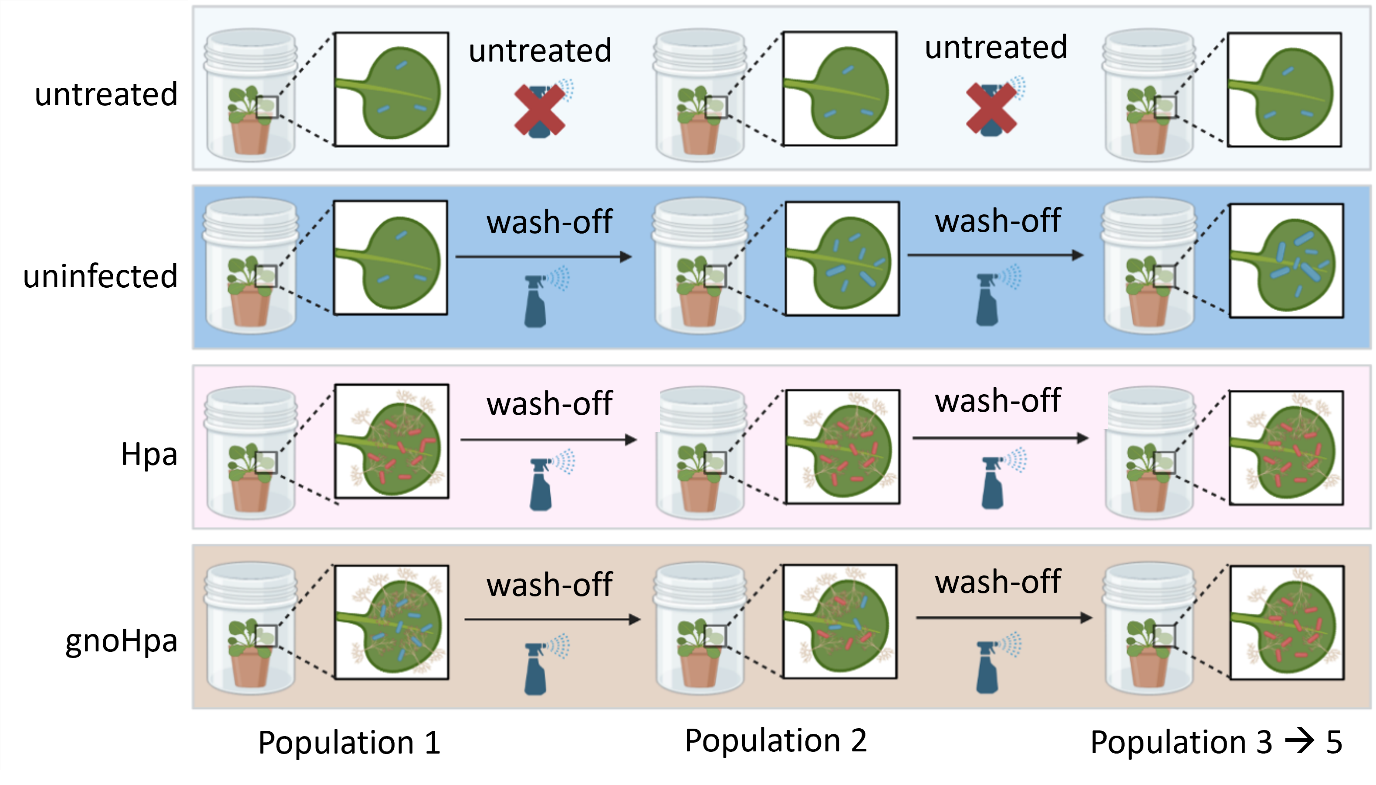
Figure S1. Passaging experimental setup.** Schematic overview of the setup used in the passaging experiment. A first plant population of Arabidopsis Col-0 plants grown in live Reijerscamp field soil was inoculated with HAM-free gnotobiotic Hpa spores (gnoHpa), regular HAM-containing Hpa spore suspensions (Hpa), a control inoculum of sterile water (uninfected) or remained untreated. From uninfected-, Hpa-, and gnoHpa-lineages, phyllosphere microbial leaf wash-offs were weekly passaged onto successive populations of freshly grown Col-0 plants. This passaging procedure was repeated for a total of five populations. From every population, phyllospheres of untreated plants were sampled as control. We hypothesized that core-HAM (indicated as red bacteria) will specifically accumulate in gnoHpa lineages whereas non-core-HAM (indicated as blue bacteria) will not.

**
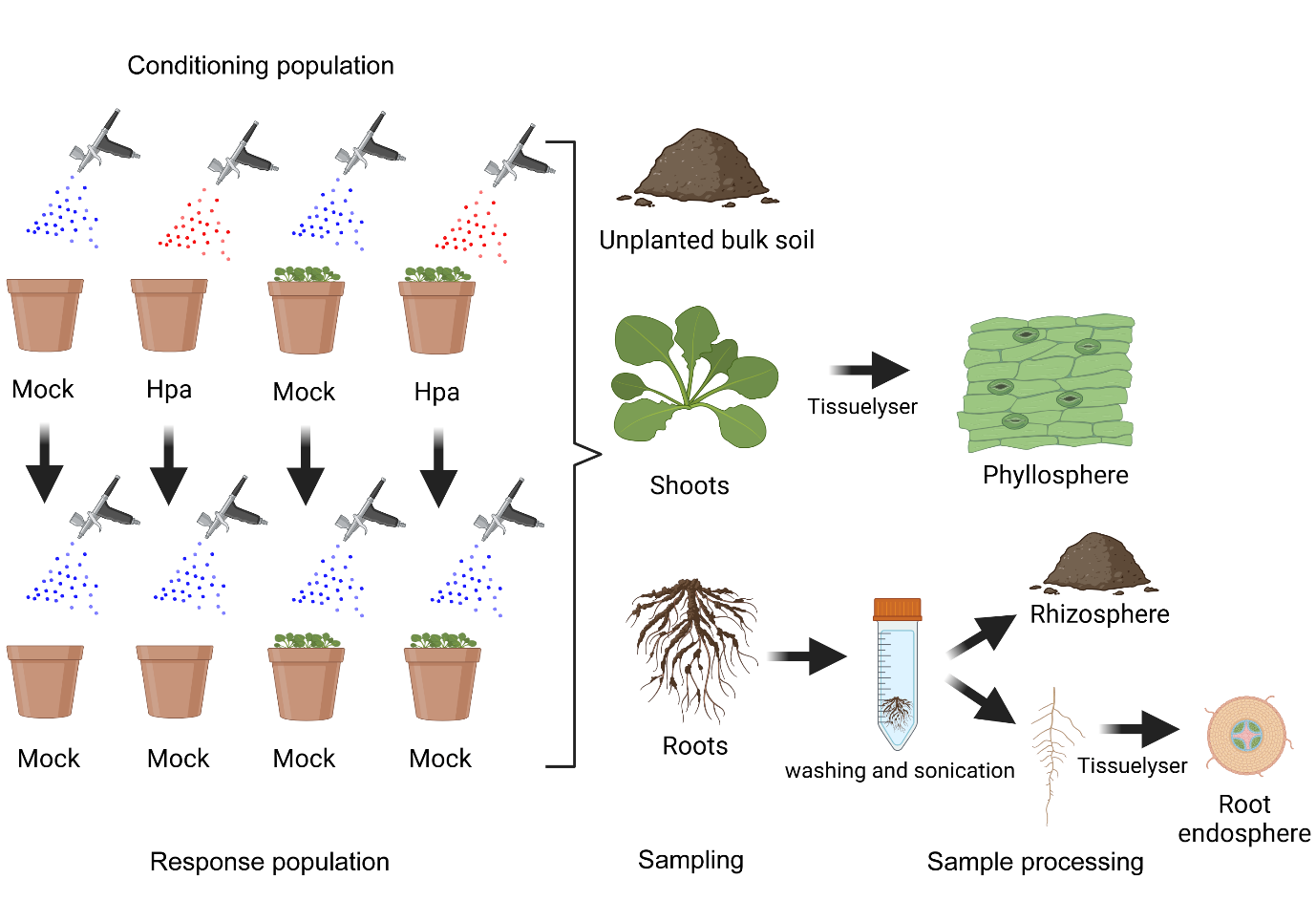
**

**Figure S2. Microbiome compartment experimental setup.** Experimental setup and sampling strategy of the microbiome compartment experiment. A conditioning population of 14 day old Arabidopsis Col-0 plants, grown in Reijerscamp field soil that possesses live microbiota, was either mock or Hpa inoculated. All above ground plant parts were removed 7 days post inoculation, and a response plant population of Arabidopsis Col-0 plants was directly sown in the mock- and Hpa-conditioned control and SBL soils, respectively. The response plant populations grown in soils conditioned by mock- or Hpa-inoculated plants were mock-treated at 14 days and harvested at 21 days after sowing. Additionally, unplanted soils were similarly treated throughout the experiment. For both the conditioning and response plant populations, root and shoot material was sampled 7 days post inoculation. Moreover, unplanted soils were sampled and considered as the bulk soil microbiome compartment. Frozen shoot material was lysed and considered the phyllosphere microbiome compartment. Roots with adhering soil were washed and sonicated. The wash-off was collected and considered the rhizosphere microbiome compartment, whereas the clean roots were frozen and lysed, and considered the root endosphere microbiome compartment.

**
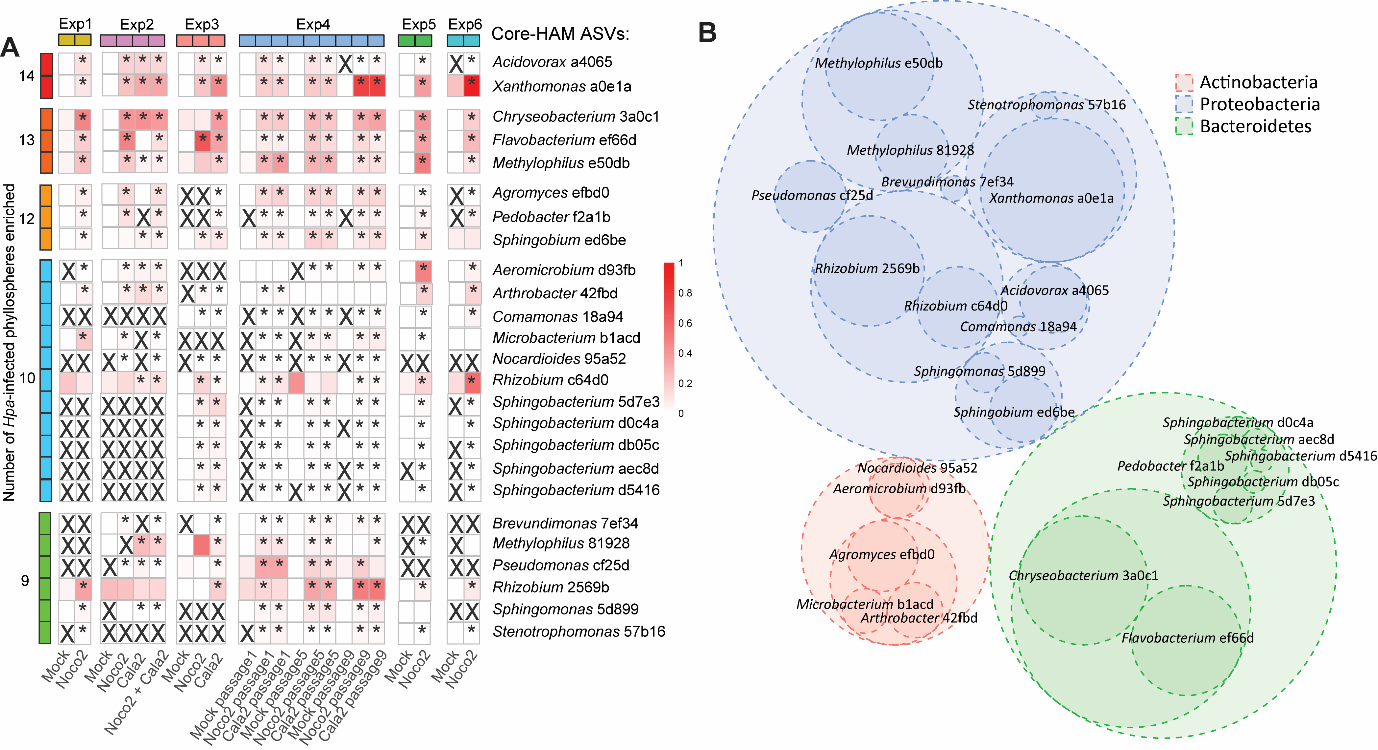
Figure S3. Abundance and taxonomic diversity of core-HAM ASVs in Hpa-infected and healthy plants across 6 independent experiments. (A)** Abundances of the 25 core-HAM ASVs that were significantly enriched (DESeq2) in more than 8 out of 14 Hpa-infected phyllospheres compared to healthy plants across 6 independent experiments, in Hpa isolate Noco2 or Cala2 infected phyllospheres or their corresponding uninfected controls. Colors represent ASV abundances per treatment scaled relatively from the most abundant (1) to least abundant (0) observation. X indicates that the ASV was not detected. ASVs are labeled by their genus and a 5 character abbreviation of their corresponding ASV ID. ASVs are ranked from top to bottom based on the number of treatments in which they were found to be significantly enriched, as indicated by *. **(B)** Bubble plot showing the taxonomic composition of the 25 member core-HAM community. Circles represent the taxonomic level from the color-coded phylum level (outer circle) to the genus level (inner circle). The circle-sizes correspond to the average relative abundance of each taxonomic level in the Hpa-infected phyllosphere across experiments. ASVs are labeled by their genus and a 5 character abbreviation of their corresponding ASV ID.

**
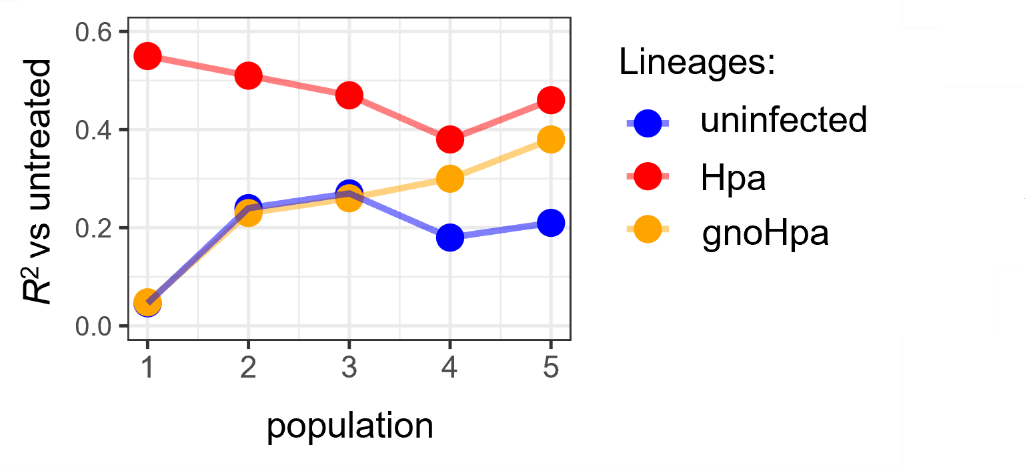
**

**Figure S4. Changes in phyllosphere microbiome composition in passaged lineages compared to untreated control plants.** Effect size of changes in phyllosphere microbiome composition, indicated by the *R*^2^ value in PERMANOVA analysis based on Bray-Curtis dissimilarites, in uninfected, gnoHpa and Hpa lineages compared to the untreated control within each Arabidopsis Col-0 plant population from the passaging experiment. Except for uninfected and gnoHpa-treated plants in population 1, all effect sizes are supported by statistically significant differences (*P* < 0.001). PERMANOVA results of all pairwise comparisons are indicated in Table S2.

**
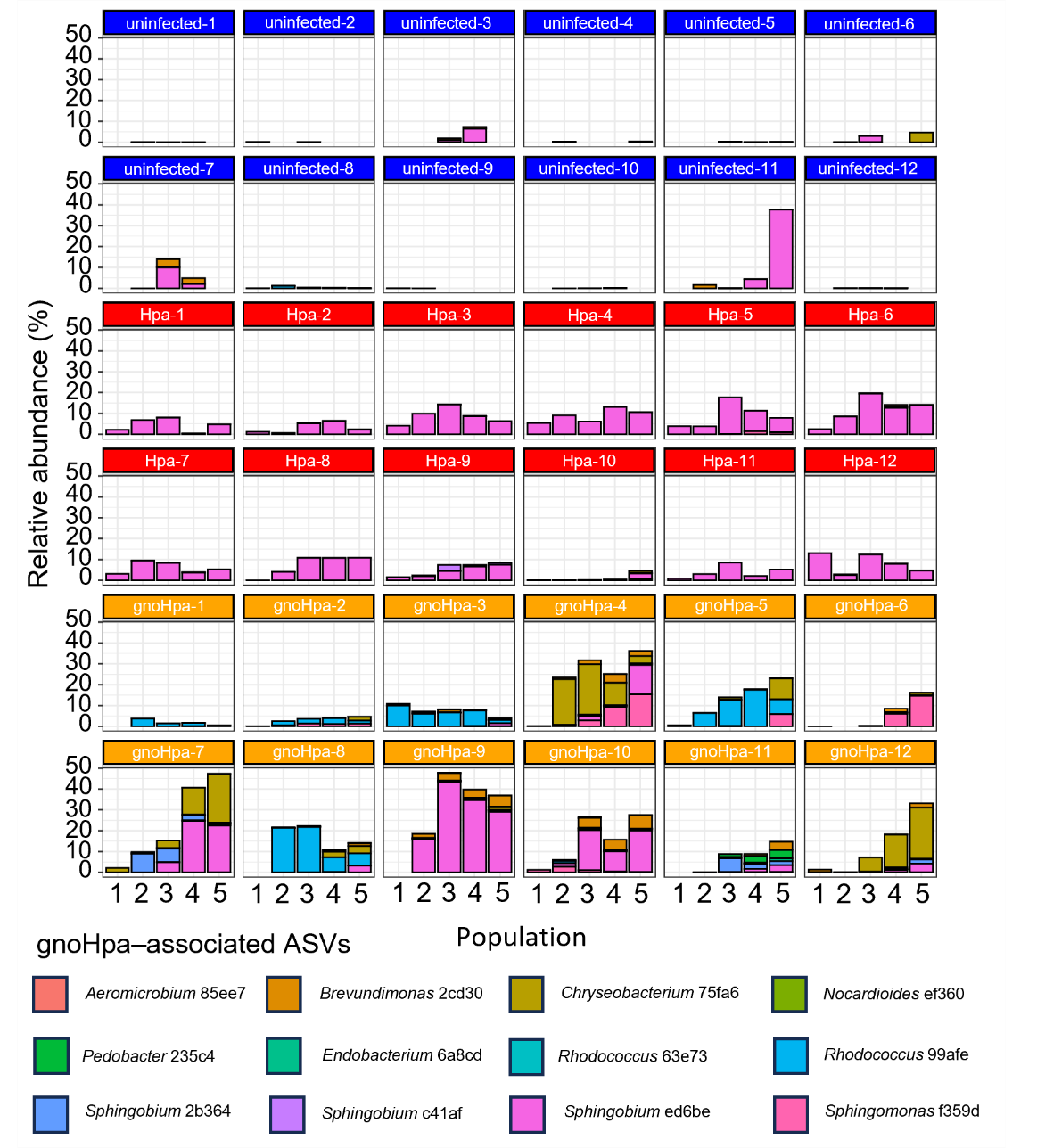
**

**Figure S5. Relative abundance of the 12 gnoHpa-associated ASVs per lineages.** Cumulative relative abundance of the 12 gnoHpa-associated ASVs in independent uninfected (blue), gnoHpa-infected (orange) or Hpa-infected (red) replicate lineages per Arabidopsis Col-0 plant population. Each treatment consists of 12 independent biological replicates, except for untreated population 1 (*N* = 11), uninfected population 4 (*N* = 11) and uninfected population 5 (*N* = 7). Each color specifically corresponds to one of the 12 ASVs that was identified as gnoHpa-associated based on the criteria that they are consistenly enriched in gnoHpa lineages compared to uninfected lineages, accumulate in gnoHpa lineages but not in uninfected lineages, and correlate to levels of downy mildew as quantified by qPCR.

**
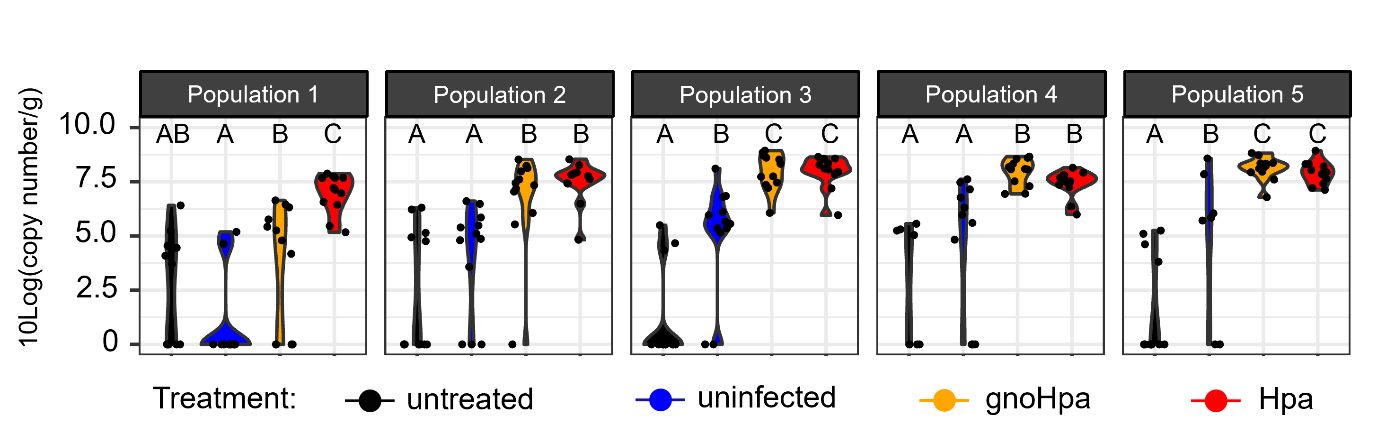
**

**Figure S6. Absolute abundances of the 12 gnoHpa-associated ASVs.** Violin plot showing the cumulative absolute abundances of the 12 gnoHpa-associated ASVs, as quantified by spike-in of *S. ruber* DNA and represented as the 10-Log 16S copy number per gram of shoot tissue, in the phyllosphere of untreated controls (black), or uninfected (blue), gnoHpa-infected (orange) or Hpa-infected (red) lineages of Arabidopsis Col-0 plant populations. Letters indicate significance level (*P* < 0.05, ANOVA with Tukey’s post-hoc test) of 12 independent biological replicates, except for untreated population 1 (*N* = 11), uninfected population 4 (*N* = 11) and uninfected population 5 (*N* = 7).

**
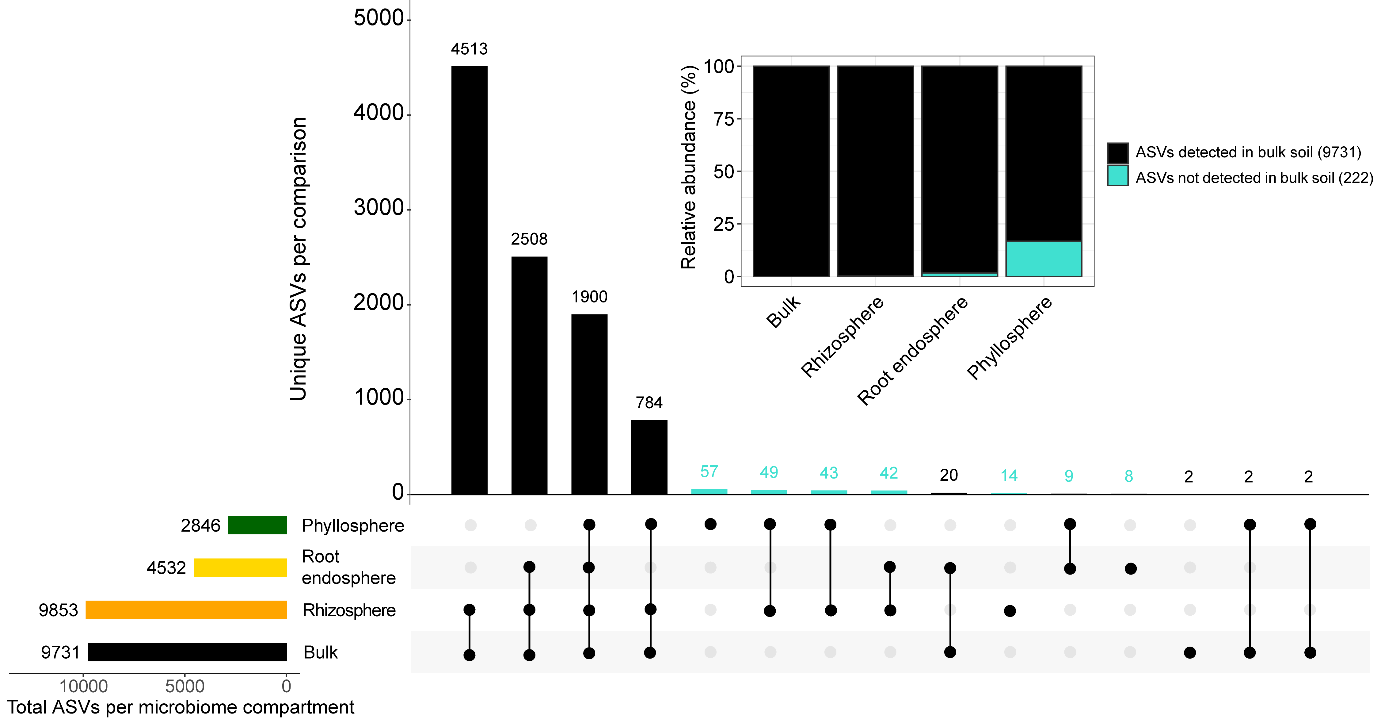
**

**Figure S7. The total of 9953 ASVs and their detection per microbiome compartment.** Upset plot showing the overlap of detected ASVs between distinct microbiome compartments. Horizontal bars represent the total number of ASVs detected per microbiome compartment. Vertical bars represent the number of ASVs unique to or shared between microbiome compartments, as indicated by the connected dots below. The 9731 ASVs that are detected in the bulk soil are represented in black, whereas the 222 ASVs that were below detection limit or absent in bulk soil were are represented in turquoise. Stacked barplots show the relative abundances of bulk soil detected and bulk soil absent ASVs per microbiome compartment.

**
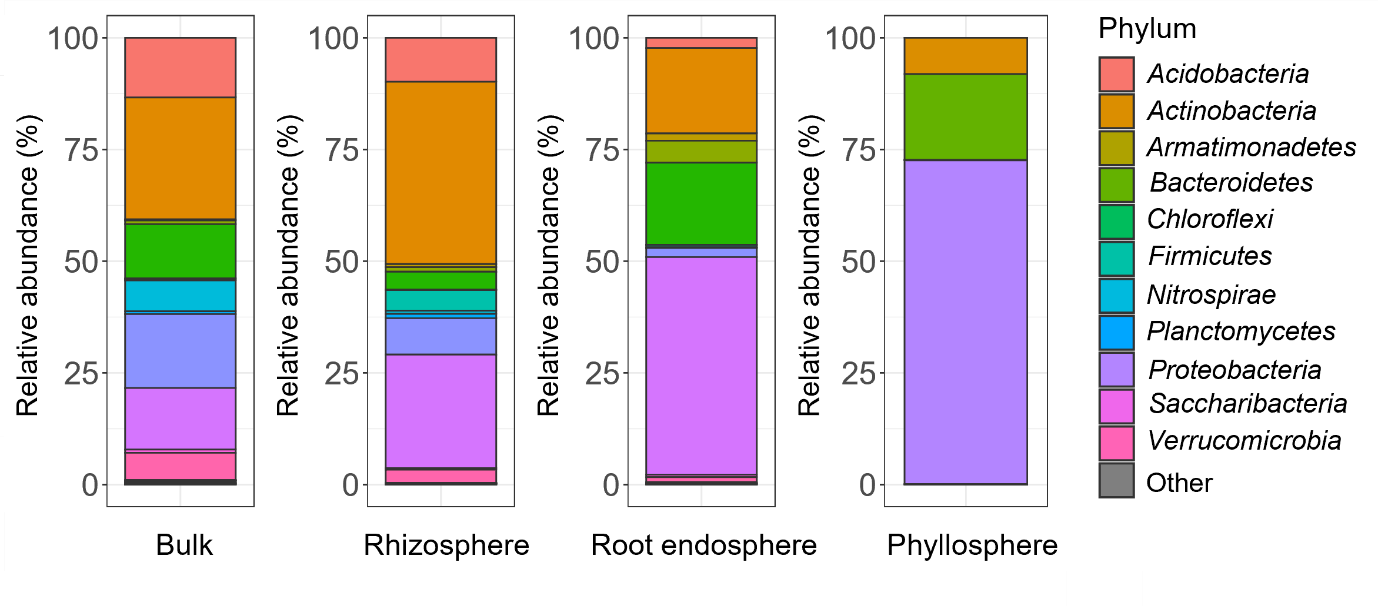
**

**Figure S8. Taxonomic diversity of ASVs that were most abundantly detected in either bulk soil, rhizosphere, root endosphere or phyllosphere microbiome compartments.** Stacked barplots showing the taxonomic distribution at the phylum level of ASVs that were most abundant in unplanted bulk soil, rhizosphere, root endosphere or phyllosphere samples, as quantified by their relative abundances within that respective microbiome compartment. Colors indicate the 11 most abundant phyla that on average cover over 0.1% relative abundance across the entire dataset.x


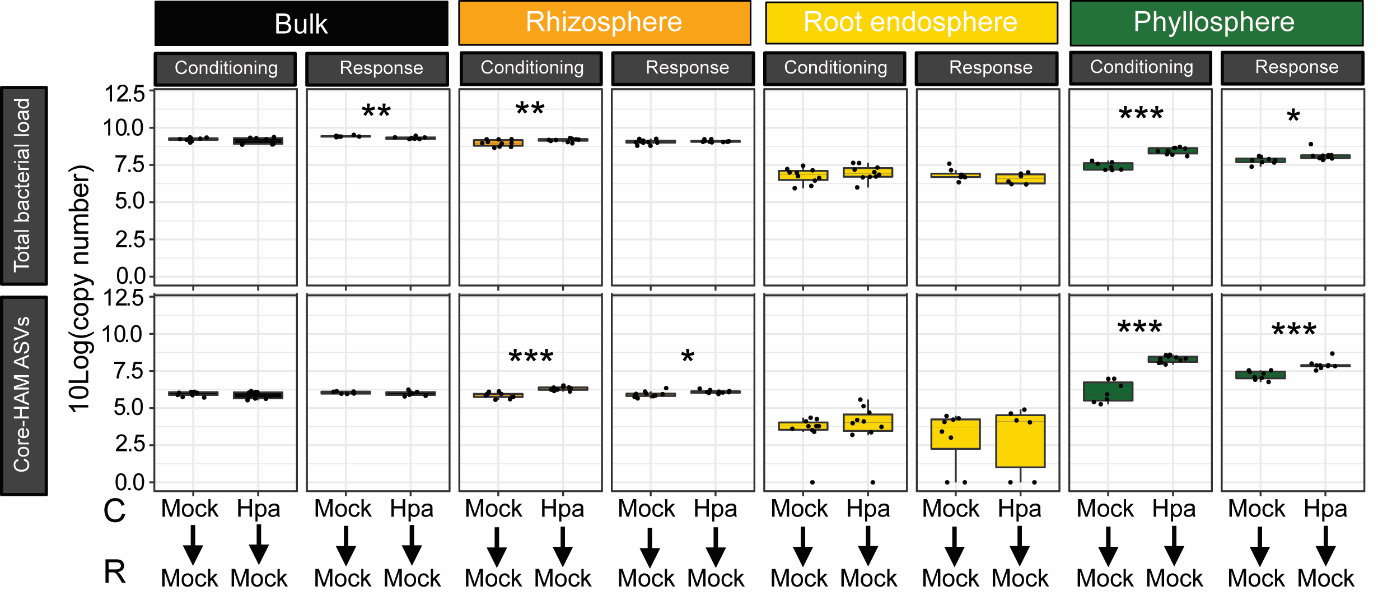


**Figure S9. Absolute abundances of the total bacterial communities and core-HAM in the microbiome compartment experiment.** Boxplots showing the absolute bacterial load and core-HAM ASVs abundances per microbiome compartment quantified via spiked-in *S. ruber* DNA. Absolute abundances are represented as Log-10 transformed 16S copy numbers. Asterisk indicate significant differences in FDR-corrected one-sided Student’s *t*-test. Total bacterial load from left to right: ***P* = 0.0073, **P* = 0.023, ****P* = 2.0 x 10^-7^, **P* = 0.024. Core-HAM from left to right ****P* = 1.6 x 10^-5^, * *P* = 0.026, ****P* = 6.5 x 10^-7^, ****P* = 8.0 x 10^-4^.

**
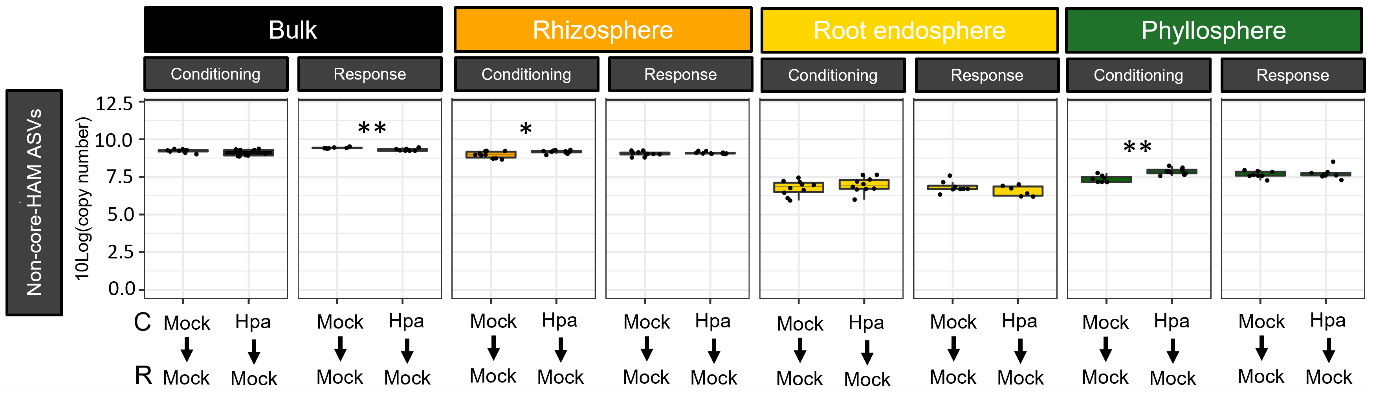
**

**Figure S10. Absolute abundances of all ASVs that are not part of the core-HAM community in the microbiome compartment experiment.** Boxplots showing absolute abundances, as quantified by spiked-in *S. ruber* DNA and represented as Log-10 transformed 16S copy numbers, of ASVs that are not part of the core-HAM community (non-core-HAM ASVs) in unplanted bulk soil, rhizosphere, root endosphere and phyllosphere microbiome compartments. Arabidopsis Col-0 plants were grown as mock- or Hpa-inoculated conditioning (C) plant populations or mock-inoculated response (R) plant populations growing in the soils conditioned by mock- or Hpa-inoculated plants. Asterisks indicate significance in FDR-corrected one-sided Student’s *t*-test of 6-10 biological replicates, from left to right: ***P* = 0.0015; **P* = 0.023; ***P* = 0.0073.

**
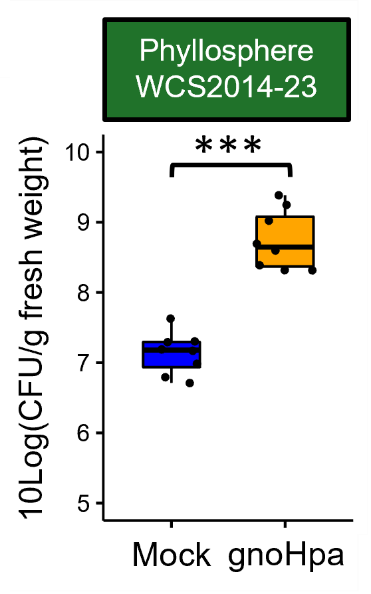
**

**Figure S11. Bacterial population densities of *Xanthomonas* isolate WCS2014-23 when co-inoculated with gnoHpa directly in the phyllosphere**. Boxplots showing abundances of *Xanthomonas* isolate WCS2014-23, represented by *Xanthomonas* HAM ASV a0e1a, upon inoculation into the phyllosphere directly in absence and presence of gnotobiotic Hpa (gnoHpa) spores. Bacterial abundance was quantified as the 10-Log of colony forming units (CFU) per gram of shoot fresh weight through serial dilution plating. Asterisks indicate significance level in one-sided Student’s *t-*test: ****P* = 2.20 x 10^-7^.

**Table S1: Core-HAM ASVs that were enriched (FDR-corrected Wald-test, DESeq2) in more than 8 out of 14 Hpa-cultures tested across 6 independent experiments that were performed in a time span of over 5 years. Taxonomy indicates genus or family level of each ASV. ASVs are depicted by the first five characters of their identifiers.**

| **Taxonomy** | **ASV ID** | **Enriched in number of cultures** |
| --- | --- | --- |
| *Acidovorax* | a4065 | 14 |
| *Xanthomonas* | a0e1a | 14 |
| *Chryseobacterium* | 3a0c1 | 13 |
| *Flavobacterium* | ef66d | 13 |
| *Methylophilus* | e50db | 13 |
| *Agromyces* | efbd0 | 12 |
| *Pedobacter* | f2a1b | 12 |
| *Sphingobium* | ed6be | 12 |
| *Aeromicrobium* | d93fb | 10 |
| *Arthrobacter* | 42fbd | 10 |
| *Comamonas* | 18a94 | 10 |
| *Microbacterium* | b1acd | 10 |
| *Nocardioides* | 95a52 | 10 |
| *Rhizobium* | c64d0 | 10 |
| *Sphingobacterium* | 5d7e3 | 10 |
| *Sphingobacterium* | aec8d | 10 |
| *Sphingobacterium* | d0c4a | 10 |
| *Sphingobacterium* | d5416 | 10 |
| *Sphingobacterium* | db05c | 10 |
| *Brevundimonas* | 7ef34 | 9 |
| *Methylophilus* | 81928 | 9 |
| *Pseudomonas* | cf25d | 9 |
| *Rhizobium* | 2569b | 9 |
| *Sphingomonas* | 5d899 | 9 |
| *Stenotrophomonas* | 57b16 | 9 |

**Table S2: PERMANOVA (permutations = 9999) results of pairwise comparisons between uninfected, Hpa and gnoHpa lineages and untreated control plants per population in the passaging experiment. *R*^2^ is a measure of effect size, *P*_adj_ represents FDR*-*corrected *P* value.**

|  | **Population 1** | | **Population 2** | | **Population 3** | | **Population 4** | | **Population 5** | |
| --- | --- | --- | --- | --- | --- | --- | --- | --- | --- | --- |
| **Treatment** | ***R*^2^** | ***P*_adj_** | ***R*^2^** | ***P*_adj_** | ***R*^2^** | ***P*_adj_** | ***R*^2^** | ***P*_adj_** | ***R*^2^** | ***P*_adj_** |
| untreated vs uninfected | 0.046 | 0.53 | 0.24 | 0.00012 | 0.27 | 0.00012 | 0.18 | 0.00012 | 0.21 | 0.00015 |
| untreated vs gnoHpa | 0.048 | 0.53 | 0.23 | 0.00012 | 0.26 | 0.00012 | 0.30 | 0.00012 | 0.38 | 0.00015 |
| untreated vs Hpa | 0.55 | 0.00020 | 0.51 | 0.00012 | 0.47 | 0.00012 | 0.38 | 0.00012 | 0.46 | 0.00015 |
| uninfected vs gnoHpa | 0.039 | 0.66 | 0.035 | 0.71 | 0.070 | 0.046 | 0.087 | 0.0075 | 0.10 | 0.0094 |
| uninfected vs Hpa | 0.50 | 0.00020 | 0.52 | 0.00012 | 0.54 | 0.00012 | 0.30 | 0.00012 | 0.36 | 0.00024 |
| gnoHpa vs Hpa | 0.52 | 0.00020 | 0.49 | 0.00012 | 0.50 | 0.00012 | 0.41 | 0.00012 | 0.47 | 0.00015 |

**Table S3: ASVs that were enriched (ANCOM-BC or DESeq2, FDR*-*corrected *P* value < 0.05) in gnoHpa-infected lineages compared to uninfected lineages in at least 2 populations of populations 2-5. Taxonomy indicates genus or family level of each ASV. ASVs are depicted by the first five characters of their identifiers.**

| **Taxonomy** | **ASV ID** | **Populations enriched** |
| --- | --- | --- |
| *Rhodococcus* | 99afe | 2, 3, 4, 5 |
| *Sphingomonas* | f359d | 2, 3, 4, 5 |
| *Aeromicrobium* | 85ee7 | 2, 3, 4, 5 |
| *Candidatus Alysiosphaera* | 732a1 | 2, 3, 4, 5 |
| *Methylobacterium* | 57701 | 2, 3, 4 |
| *Caulobacter* | fc672 | 2, 3, 4 |
| *Methylobacterium* | a0ba4 | 2, 3, 5 |
| *Rhodococcus* | 8c5b0 | 2, 3, 5 |
| *Chryseobacterium* | 75fa6 | 2, 3, 5 |
| *Stenotrophomonas* | 74299 | 2, 3, 5 |
| *uncultured bacterium* | 1e488 | 2, 3, 5 |
| *Burkholderia-Paraburkholderia* | e8fde | 2, 3, 5 |
| *Paenarthrobacter* | feefd | 2, 3, 5 |
| *Bosea* | b24aa | 2, 3, 5 |
| *Bacillus* | e4613 | 2, 3, 5 |
| *Bacillus* | 253a7 | 2, 3, 5 |
| *Sphingobium* | 2b364 | 3, 4, 5 |
| *Marmoricola* | eb644 | 3, 4, 5 |
| *Curtobacterium* | cfa64 | 3, 4, 5 |
| *Mycobacterium* | f51ee | 3, 4, 5 |
| *Endobacterium* | 6a8cd | 3, 4, 5 |
| *Sphingobium* | c41af | 3, 4, 5 |
| *Methylobacterium* | 4715d | 3, 4, 5 |
| *Leifsonia* | 0bb97 | 2, 3 |
| *Serratia* | e8165 | 2, 3 |
| *Enterobacter* | 32ab1 | 2, 3 |
| *Brevundimonas* | b679f | 2, 3 |
| *Nocardioides* | ad301 | 2, 3 |
| *Mucilaginibacter* | 58ed4 | 2, 3 |
| *Blastococcus* | dcda0 | 2, 3 |
| *Nocardioidaceae* | 90a9f | 2, 3 |
| *Rhodococcus* | 599b0 | 2, 3 |
| *Nocardia* | 03cc1 | 2, 3 |
| *Burkholderia-Paraburkholderia* | 1bd14 | 2, 3 |
| *Luteibacter* | 1eab4 | 2, 4 |
| *Methylobacterium* | 2b455 | 2, 4 |
| *Chryseobacterium* | 84207 | 2, 4 |
| *Paenibacillus* | 99fe4 | 2, 4 |
| *Rhodococcus* | 43515 | 2, 5 |
| *Sphingobium* | ed6be | 2, 5 |
| *Williamsia* | 7662b | 2, 5 |
| *Rhizobium* | 0edfe | 2, 5 |
| *Geodermatophilaceae* | 89606 | 2, 5 |
| *Nakamurella* | d90c1 | 2, 5 |
| *Burkholderia-Paraburkholderia* | b9a50 | 2, 5 |
| *Pseudomonas* | 7e5e0 | 2, 5 |
| *Rhizobium* | e405a | 2, 5 |
| *Devosia* | c7511 | 2, 5 |
| *Rhodococcus* | 63e73 | 2, 5 |
| *Nocardioides* | ed035 | 2, 5 |
| *Actinomycetospora* | 9a60d | 2, 5 |
| *Tepidisphaeraceae* | be9ff | 2, 5 |
| *Bosea* | a756b | 3, 4 |
| *Rhizomicrobium* | d1a41 | 3, 4 |
| *Variovorax* | 47082 | 3, 4 |
| *Brevundimonas* | 2cd30 | 3, 5 |
| *Mycobacterium* | d6c91 | 3, 5 |
| *Nocardioides* | ed3d4 | 3, 5 |
| *uncultured bacterium* | 70a25 | 3, 5 |
| *Sphingomonas* | cc397 | 3, 5 |
| *Microbacteriaceae* | 8fc0f | 3, 5 |
| *Burkholderia-Paraburkholderia* | 64475 | 3, 5 |
| *Mycobacterium* | 9f811 | 3, 5 |
| *Leifsonia* | 3b427 | 3, 5 |
| *Mycobacterium* | bf19f | 3, 5 |
| *Nakamurella* | 3c922 | 3, 5 |
| *Methylobacterium* | 31c5d | 4, 5 |
| *Caulobacter* | 52d6f | 4, 5 |
| *Pedobacter* | 235c4 | 4, 5 |
| *Microbacterium* | f0c76 | 4, 5 |
| *Nocardioides* | ef360 | 4, 5 |
| *Paenibacillus* | b454b | 4, 5 |
| *Blastococcus* | 65651 | 4, 5 |
| *Mycobacterium* | 63be8 | 4, 5 |
| *Paenarthrobacter* | 844b8 | 4, 5 |

**Table S4: ASVs that were enriched (ANCOM-BC or DESeq2, FDR*-*corrected *P* value < 0.05) in successive populations within gnoHpa-lineages compared to population 1, but not in uninfected-lineages. Taxonomy indicates genus, family or order level of each ASV. ASVs are depicted by the first five characters of their identifiers.**

| **Taxonomy** | **ASV ID** | **Populations enriched** |
| --- | --- | --- |
| *Rhodococcus* | 99afe | 2, 3, 4, 5 |
| *Rhizobium* | b3d3c | 2, 3, 4, 5 |
| *Blastococcus* | 65651 | 2, 3, 4, 5 |
| *Brevundimonas* | 2cd30 | 2, 3, 4, 5 |
| *Chryseobacterium* | 75fa6 | 2, 3, 4, 5 |
| *Sphingobium* | ed6be | 2, 3, 4, 5 |
| *Bacillus* | df84f | 2, 3, 4, 5 |
| *Methylobacterium* | 31c5d | 2, 3, 4, 5 |
| *Nakamurella* | d90c1 | 2, 3, 4, 5 |
| *Sphingomonas* | f359d | 2, 3, 4, 5 |
| *Aeromicrobium* | 85ee7 | 2, 3, 4, 5 |
| *Rhodococcus* | 63e73 | 2, 3, 4, 5 |
| *Paenarthrobacter* | cb322 | 2, 3, 4, 5 |
| *Paenarthrobacter* | 844b8 | 2, 3, 4, 5 |
| *Hyphomicrobium* | b1334 | 3, 4, 5 |
| *Sphingobium* | 2b364 | 3, 4, 5 |
| *Curtobacterium* | cfa64 | 3, 4, 5 |
| *Xanthobacteraceae* | 3e90e | 3, 4, 5 |
| *Microbacterium* | f0c76 | 3, 4, 5 |
| *Endobacterium* | 6a8cd | 3, 4, 5 |
| *Sphingobium* | c41af | 3, 4, 5 |
| *uncultured bacterium* | ff45f | 3, 4, 5 |
| *Tardiphaga* | a57b5 | 3, 4, 5 |
| *Mycobacterium* | 63be8 | 3, 4, 5 |
| *Mucilaginibacter* | 617e9 | 3, 4, 5 |
| *Caulobacter* | 52d6f | 4, 5 |
| *Pedobacter* | 235c4 | 4, 5 |
| *Pseudomonas* | 8be20 | 4, 5 |
| *Nocardioides* | ef360 | 4, 5 |
| *Rhizobiales* | 94335 | 4, 5 |

**Table S5: ASVs that correlate (spearman, FDR-corrected *P* value < 0.05, indicated by ‘*P*_adj_’) with gnoHpa disease-quantification by qPCR compared to uninfected-lineages. *R*^2^ is a measure for effect size. Taxonomy indicates genus or family level of each ASV. ASVs are depicted by the first five characters of their identifiers.**

| **Taxonomy** | **ASV ID** | ***R*^2^** | ***P*_adj_** |
| --- | --- | --- | --- |
| *Chryseobacterium* | 75fa6 | 0.54 | 3.15E-07 |
| *Rhodococcus* | 8c5b0 | 0.52 | 5.80E-07 |
| *Rhodococcus* | 99afe | 0.51 | 1.15E-06 |
| *Brevundimonas* | 2cd30 | 0.45 | 4.44E-05 |
| *Sphingobium* | 2b364 | 0.43 | 1.07E-04 |
| *Sphingobium* | ed6be | 0.40 | 5.00E-04 |
| *Sphingobium* | c41af | 0.38 | 1.79E-03 |
| *Microbacteriaceae* | 8fc0f | 0.37 | 1.79E-03 |
| *Aeromicrobium* | 85ee7 | 0.37 | 1.79E-03 |
| *Rhodococcus* | 9c8fe | 0.35 | 4.05E-03 |
| *Sphingomonas* | f359d | 0.35 | 4.92E-03 |
| *Paenibacillus* | f5d2a | 0.35 | 4.92E-03 |
| *Endobacterium* | 6a8cd | 0.34 | 4.92E-03 |
| *Stenotrophomonas* | 74299 | 0.34 | 4.96E-03 |
| *Variovorax* | 47082 | 0.32 | 1.13E-02 |
| *Nocardioides* | ef360 | 0.32 | 1.15E-02 |
| *Pedobacter* | 235c4 | 0.31 | 1.68E-02 |
| *Methylobacterium* | 4715d | 0.31 | 1.87E-02 |
| *Pedobacter* | 48999 | 0.30 | 2.16E-02 |
| *Methylobacterium* | a0ba4 | 0.30 | 2.43E-02 |
| *Pedobacter* | fe6b9 | 0.28 | 3.99E-02 |
| *Pseudomonas* | 8be20 | 0.28 | 3.99E-02 |
| *Rhodococcus* | 63e73 | 0.27 | 4.89E-02 |

**Table S6: PERMANOVA (permutations = 9999) of factors ‘Compartment’ * ‘Treatment’ * ‘Generation’ on Bray-Curtis dissimilarities. Compartment refers to the bulk soil, rhizosphere, root endosphere and phyllosphere, Treatment to mock and Hpa inoculation and ‘Generation’ to conditioning and response population plants. *R*^2^ is a measure of effect size.**

| **Factor** | ***R*^2^** | ***P* value** |
| --- | --- | --- |
| Compartment | 0.57 | 0.0001 |
| Treatment | 0.018 | 0.0003 |
| Generation | 0.0072 | 0.019 |
| Compartment * Treatment | 0.055 | 0.0001 |
| Treatment * Generation | 0.0047 | 0.082 |
| Compartment * Generation | 0.018 | 0.0045 |
| Compartment * Treatment * Generation | 0.015 | 0.023 |
| Residual | 0.31 | NA |

**Table S7: PERMANOVA (permutations = 9999) results of pairwise comparisons between distinct microbiome compartments. *R*^2^ is a measure of effect size, *P*_adj_ represents FDR*-*corrected *P* value.**

| **Group 1** | **Group 2** | ***R*^2^** | ***P*_adj_** |
| --- | --- | --- | --- |
| Bulk | Rhizosphere | 0.10 | 0.0001 |
| Bulk | Root Endosphere | 0.47 | 0.0001 |
| Bulk | Phyllosphere | 0.57 | 0.0001 |
| Rhizosphere | Root Endosphere | 0.48 | 0.0001 |
| Rhizosphere | Phyllosphere | 0.59 | 0.0001 |
| Root endosphere | Phyllosphere | 0.32 | 0.0001 |

**Table S8: PERMANOVA (permutations = 9999) results of treatment effect (Mock vs Hpa) within microbiome compartments in conditioning population plants and response population plants. *R*^2^ is a measure of effect size.**

|  | **Conditioning plant population** | | **Response plant population** | |
| --- | --- | --- | --- | --- |
| **Compartment** | ***R*^2^** | ***P* value** | ***R*^2^** | ***P* value** |
| Bulk | 0.049 | 0.83 | 0.13 | 0.0002 |
| Rhizosphere | 0.069 | 0.011 | 0.081 | 0.0005 |
| Root endosphere | 0.056 | 0.29 | 0.079 | 0.34 |
| Phyllosphere | 0.52 | 0.0001 | 0.35 | 0.0001 |

**Table S9: Core-HAM ASVs and detection (+/-) in the phyllosphere, rhizosphere, root endosphere and bulk soil Taxonomy indicates genus or family level of each ASV. ASVs are depicted by the first five characters of their identifiers.**

| **Taxonomy** | **ASV ID** | **Phyllosphere** | **Rhizosphere** | **Root endosphere** | **Bulk soil** |
| --- | --- | --- | --- | --- | --- |
| *Acidovorax* | a4065 | + | - | - | - |
| *Xanthomonas* | a0e1a | + | + | + | + |
| *Chryseobacterium* | 3a0c1 | + | + | + | + |
| *Flavobacterium* | ef66d | + | + | + | + |
| *Methylophilus* | e50db | + | + | + | + |
| *Agromyces* | efbd0 | + | - | - | - |
| *Pedobacter* | f2a1b | + | + | + | - |
| *Sphingobium* | ed6be | + | + | + | + |
| *Aeromicrobium* | d93fb | + | + | + | - |
| *Arthrobacter* | 42fbd | + | + | + | + |
| *Comamonas* | 18a94 | + | + | - | - |
| *Microbacterium* | b1acd | + | + | - | - |
| *Nocardioides* | 95a52 | - | - | - | - |
| *Rhizobium* | c64d0 | + | + | + | + |
| *Sphingobacterium* | 5d7e3 | + | + | - | - |
| *Sphingobacterium* | aec8d | + | - | - | - |
| *Sphingobacterium* | d0c4a | + | + | - | - |
| *Sphingobacterium* | d5416 | + | - | - | - |
| *Sphingobacterium* | db05c | + | - | - | - |
| *Brevundimonas* | 7ef34 | - | - | - | - |
| *Methylophilus* | 81928 | + | - | + | + |
| *Pseudomonas* | cf25d | - | - | - | - |
| *Rhizobium* | 2569b | + | + | - | - |
| *Sphingomonas* | 5d899 | - | - | - | - |
| *Stenotrophomonas* | 57b16 | + | + | - | - |
